# The Heart’s Small Molecules: The Importance of MicroRNAs in Cardiovascular Health

**DOI:** 10.3390/jcm14217454

**Published:** 2025-10-22

**Authors:** Mustafa Yildiz, Ugur Ozkan, Metin Budak

**Affiliations:** 1 Department of Biophysics, Faculty of Medicine, Trakya University, 22030 Edirne, Türkiye; 2 Department of Cardiology, Faculty of Medicine, Trakya University, 22030 Edirne, Türkiye

**Keywords:** microRNAs, cardiovascular diseases, heart failure, arrhythmias, ischemia, hypertrophy, coronary artery disease, congenital heart disease, valvular heart disease, cardiomyopathy

## Abstract

This comprehensive review explores the critical roles of microRNAs (miRNAS) in cardiovascular diseases, emphasizing their regulatory functions in gene expression and their involvement in disease progression. miRNAS are small, evolutionarily conserved non-coding RNAs that regulate gene expression post-transcriptionally and play essential roles in various cardiac conditions, including fibrosis, cardiac remodeling, apoptosis, ischemia/reperfusion injury, hypertrophy, heart failure, arrhythmias, coronary artery disease (CAD), congenital heart diseases (CHDs), cardiomyopathies, and valvular heart disease (VHD). miRNAS are increasingly recognized as sensitive and specific biomarkers for early diagnosis, disease monitoring, and evaluation of therapeutic responses across the cardiovascular disease spectrum. Ischemia/reperfusion injury leads to significant cardiac damage through elevated oxidative stress, mitochondrial dysfunction, and apoptosis. CAD, a major contributor to global morbidity and mortality, is primarily driven by atherosclerosis and chronic inflammation. Cardiac hypertrophy is initially an adaptive response to stress but may progress to heart failure if sustained. Arrhythmias arise from electrical disturbances such as reentry, abnormal automaticity, and triggered activity. Heart failure is a complex and progressive syndrome marked by poor prognosis and increasing global prevalence. VHD involves intricate molecular alterations, including myocardial fibrosis and calcification, which contribute to disease progression and adverse outcomes. Cardiomyopathies—including hypertrophic, dilated, restrictive, and arrhythmogenic forms—are influenced by genetic mutations, systemic diseases, and disrupted molecular signaling. CHDs, the most common congenital malformations, stem from structural abnormalities in cardiac development and remain a major cause of infant morbidity and mortality. Novel therapeutic methods, such as antisense oligonucleotides, miR mimics, and exosome-based delivery mechanisms, demonstrate the translational promise of miRNAs in the realm of personalized cardiovascular medicine. However, issues such as small sample sizes, inconsistent results, interspecies differences, and delivery challenges restrict the clinical application of miRNA-based therapies. This review integrates mechanistic insights, critiques the quality of available evidence, and identifies translational shortcomings. It highlights the diagnostic, prognostic, and therapeutic potential of miRNAs in reshaping cardiovascular disease treatment.

## 1. Introduction

**Figure 1:**
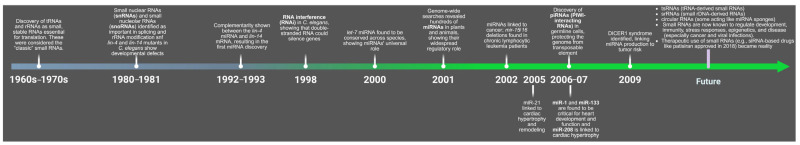
Historical milestones in small RNA research.

miRNAS are small RNA molecules that are evolutionarily conserved and play a pivotal role in the regulation of gene expression after transcription. They belong to a broader class of small RNAs involved in RNA silencing pathways and function similarly to small interfering RNAs (siRNAs). Multiple studies have indicated that miRNAs regulate diverse biological processes (extensively reviewed in [1,2,3]). Abnormal patterns of miR expression have been associated with heart disease in humans, and studies utilizing mouse models where the processing of miRNAS is specifically blocked have shown their critical importance in heart development and function [4]. Unique miRNA expression profiles associated with pathological cardiac hypertrophy, heart failure, and myocardial infarction in both humans and animal models have been summarized in the review [5]. Original studies supporting these associations are presented below. First discovered in 1993 in nematodes and initially thought to exist only during development, miRNAs have since been found in many species and shown to affect physiological functions in adults (Figure 1) [6].

**Figure 2:**
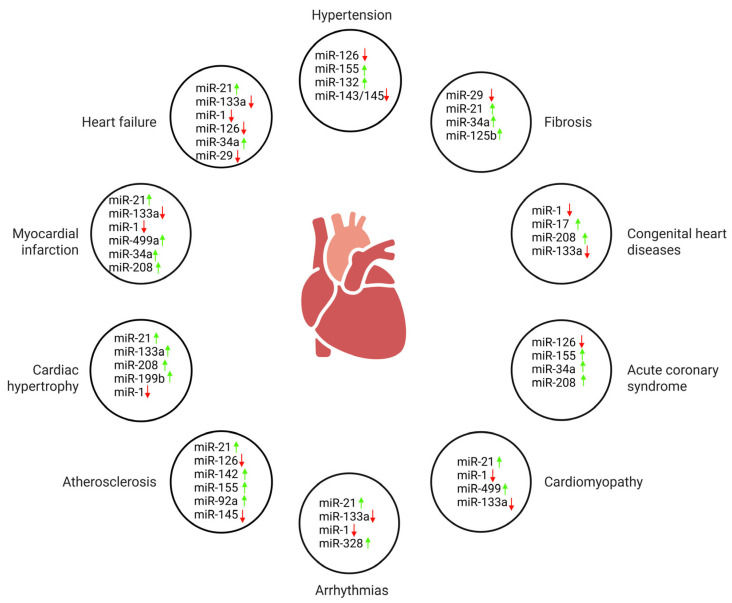
Representative microRNAs associated with major cardiovascular conditions. These reflect the most frequently observed and broadly recognized associations in the literature but do not encompass all miRNAs described in each condition. (↑) indicate upregulated and (↓) indicate downregulated miRNAs.

miRNAS have been recognized in virtually all biological fluids and are especially stable due to their resistance to degradation by nucleases, as pointed out in Turchinov’s review [7]. miRNAS are recognized as one of the largest and most functionally diverse families of small non-coding RNAs. They regulate gene expression by binding to complementary sequences in target mRNAs, enabling a single miR to potentially influence the expression of hundreds of genes [8]. Current estimates indicate that miRNAS have the ability to regulate up to 60% of all human genes, which allows them to impact a broad spectrum of biological processes and signaling pathways [9]. miRNAS are increasingly recognized for their roles in cardiovascular diseases, particularly heart failure. They regulate key pathological processes, such as cardiac fibrosis and hypertrophy, by targeting mRNAs (Figure 2) [10]. In addition to their intracellular regulatory functions, miRNAs can also be released into the extracellular environment as cell-free RNAs (cfRNAs), particularly from stressed or damaged cells. cfRNAs are released into bodily fluids from cells that are stressed or damaged, circulating freely or within extracellular vesicles while maintaining disease-specific signatures. Their presence in blood, urine, saliva, and cerebrospinal fluid renders them appealing as non-invasive biomarkers for cardiovascular diseases, such as atherosclerosis, thrombosis, and stroke. cfRNAs not only furnish diagnostic insights but may also play a role in disease pathology, as evidenced by cf-ribosomal RNAs, whose detrimental effects can be mitigated by RNases. The integration of cfRNA profiling with established cardiovascular biomarkers presents a promising strategy for early diagnosis, disease monitoring, and personalized management [11].

Despite significant advances in understanding miRNA functions, key gaps remain in translating these findings into clinical applications for cardiovascular diseases. Current literature often focuses on mechanistic insights from preclinical models, with limited data on human validation, standardized detection methods, and clinical trial outcomes. This review aims to synthesize the roles of miRNAs in cardiovascular diseases, critically evaluate the quality and limitations of existing evidence, and highlight their potential as diagnostic, prognostic, and therapeutic tools to address these translational gaps.

## 2. Methods

### Systematic Literature Search and Study Selection

This review was conducted in accordance with the PRISMA 2020 guidelines. A search of PubMed and Web of Science was performed up to 5 September 2025 utilizing the search string “(microRNA OR miRNA) AND (cardiovascular OR heart OR ischemia OR arrhythmia OR cardiomyopathy OR heart failure OR congenital heart disease OR valvular)” and associated terms. The primary inclusion criteria consisted of (i) studies that explicitly explored the relationship between miRNAs and at least one cardiovascular disease; (ii) original data from clinical (cohort, case–control, clinical trial) or preclinical (animal, primary cells/cell lines; in vivo/in vitro) research; and (iii) studies that assessed pathogenesis, diagnostic/prognostic biomarker relevance, or therapeutic targetability (including miRNA expression/function/variants and target pathways). Reviews and meta-analyses were referenced to inform background and evidence mapping and for backward/forward citation chasing (snowballing); however, as they do not provide primary data, they were excluded from quantitative data extraction, risk-of-bias evaluation, or any meta-analytic calculations.

The exclusion criteria included publication types that do not contain primary data (editorials, letters, case reports, abstract-only records), lack of adequate full-text access or insufficient methods/results for evaluation, absence of a cardiovascular or microRNA focus, studies limited to risk-factor associations without a defined cardiac outcome, and grey literature. Title/abstract screening followed by full-text evaluation was conducted independently by two reviewers; any disagreements were resolved by a third reviewer; duplicates were removed. Eligible studies were classified by theme: Ischemia–Apoptosis, Cardiac Arrhythmia, Cardiomyopathy, Hypertrophy, Coronary Artery Disease, Heart Failure, Congenital Heart Disease, and Valvular Disease. Data extraction was performed using a standardized form (sample size and population/model, biological material and assay, direction of miRNA change, targets/pathways and clinical/experimental endpoints such as diagnostic AUC or prognostic associations). This framework was designed to comprehensively capture preclinical and clinical evidence along the miRNA–cardiovascular axis while prioritizing methodological rigor, reproducibility, and translational relevance.

## 3. Regulation of Apoptosis in Ischemia/Reperfusion Injury by MicroRNAs

Ischemia–reperfusion injury (IRI) occurs when blood supply is restored after a period of ischemia (reduced blood flow). Reperfusion then causes additional damage via inflammation and oxidative stress [12]. The injury caused by ischemia/reperfusion (I/R) leads to extensive damage to the heart, typically resulting in a reduction or complete loss of cardiac function, thus hindering the benefits of reperfusion therapy post-acute myocardial infarction (AMI) [13]. Thus, I/R injury is a critical global health concern. I/R injury is implicated in many conditions (e.g., sleep apnea, acute kidney injury (AKI), circulatory arrest, sickle cell disease, ischemic stroke (IS), and trauma) as well as in acute myocardial infarction. Moreover, it represents a critical issue in organ transplantation and major surgical operations [14]. Mitochondria, integral to ATP production and the survival of cells, are adversely affected during ischemia–reperfusion (I/R), leading to heightened reactive oxygen species (ROS) levels and cellular damage. Mitochondrial dysfunction is a primary contributor to cardiomyopathies and injuries resulting from I/R [15,16]. These factors are critical in causing tissue damage, particularly in the brain and heart, which underscores the significance of mitochondrial function as a primary therapeutic target in ischemia–reperfusion-related diseases [17]. Despite current therapeutic options, myocardial ischemia–reperfusion injury remains a predominant factor in mortality associated with acute coronary syndrome (Table 1) [18,19].

In cardiomyocytes experiencing apoptosis due to angiotensin I (ang II), NF-κB positively regulates the expression of miR-30b, which directly targets Bcl-2 [20]. miR-24 serves to suppress apoptosis in cardiomyocytes, diminish infarct size, and improve cardiac function, primarily through the direct inhibition of Bim, a BH3-only domain protein that enhances apoptosis [26]. Inhibiting miR-15b resulted in an increase in the levels of Bcl-2 protein, with no impact on Bcl-2 mRNA levels. This change curtailed the release of mitochondrial cytochrome c into the cytosol and diminished the activities of both caspase-3 and caspase-9 [21]. The protective effect of carvedilol on cardiomyocytes is attributed to its ability to elevate miR-133 levels while simultaneously suppressing caspase-9 and the downstream apoptotic pathways [24]. Hypoxia/reoxygenation (H/R) triggered apoptosis in H9c2 cardiomyocytes, which was associated with a decrease in miR-101 expression, and revealed Ras-related protein Rab-5A (RAB5A) as a direct target of miR-101 [22]. The expression of miR-199a is markedly diminished in cardiac myocytes when oxygen tension decreases. This decrease is crucial for prompt upregulation of its target, hypoxia-inducible factor (HIF)-1α. By reintroducing miR-199a during hypoxia, the expression of HIF-1α and its stabilization of p53 are inhibited, which consequently lowers the rate of apoptosis [25].

An increase in miR-1 and miR-133a levels led to a reduction in cardiomyocyte apoptosis and miR-133a mimic was found to downregulate caspase-9 protein expression, thereby diminishing apoptosis induced by ischemia–reperfusion [28]. During the processes of I/R and H/R, induced expression of miR-21 was observed to upregulate Akt signaling activity through suppression of phosphatase and tensin homolog (PTEN). The resulting increase in Akt signaling activity played a role in partially inhibiting apoptosis by increasing the ratio of B-cell lymphoma 2 (Bcl-2) to Bcl-2-associated X protein (Bax), which further led to a decrease in caspase-3 expression [32]. miR-30 is instrumental in regulating the β-adrenergic pathway, where it combines direct inhibition of β1- and β2-adrenoceptors (β1AR and β2AR) with targeting of Giα-2 for enhanced regulation. Additionally, miR-30 targets the pro-apoptotic gene BNIP3L/NIX, and increased levels of miR-30 are protective against the toxicity of doxorubicin (DOX). The expression of miR-34a is associated with the decline in Bcl-2 levels induced by elevated glucose, which subsequently results in cardiomyocyte apoptosis [31]. In contrast, miR-1 mimics negated the protective the role of IGF-1 against glucose-induced mitochondrial dysfunction, cytochrome-c release, and apoptosis, whereas a mutant form of miR-1 had no such effect [34].

miRNAs exhibit considerable potential as biomarkers and therapeutic targets in ischemia–reperfusion injury (IRI). Increased plasma concentrations of miR-21 and miR-1 are associated with the severity of IRI following acute myocardial infarction (AMI), thereby establishing them as non-invasive prognostic biomarkers [24,36]. For example, the elevation of miR-21 in plasma correlates with negative cardiac remodeling, which could facilitate risk stratification [24]. On a therapeutic level, miR-133a mimics have been shown to decrease apoptosis in animal models, while miR-21 inhibitors alleviate inflammation and IRI damage through PTEN/Akt pathway modulation [28,32]. Endothelial dysfunction is a key factor in ischemia with non-obstructive coronary artery (INOCA), with diabetes intensifying microvascular injury. Recent findings suggest that circulating microRNAs could be effective biomarkers in this scenario. Notably, miR-363-5p and miR-92a-3p were shown to be dysregulated in individuals with diabetic INOCA, thereby linking changes in endothelial miRNA profiles to the severity of the condition [41]. These observations indicate that miRNAs may play a crucial role in shaping personalized treatment approaches. Nonetheless, the clinical application of these findings encounters several obstacles, such as ineffective delivery mechanisms, off-target effects, and prohibitive costs. A notable challenge is the targeted delivery of miRNA mimics or inhibitors to cardiomyocytes without impacting surrounding tissues. Presently, clinical trials are scarce, with miR-21 inhibitors in the preliminary stages of development for cardiovascular uses, yet comprehensive human data remain insufficient. Innovative delivery methods, including nanoparticle-based systems, hold promise for overcoming these challenges but necessitate further validation.

The apoptosis of cardiomyocytes in response to ischemia, hypoxia and oxidative stress is reliably regulated by a small group of miRNAS that show translational promise. In both cellular and various in vivo rodent models, miR-21 (via PDCD4 and PTEN/Akt), miR-24 (Bim), miR-133a (Casp9), miR-30 (p53–Drp1 and β-adrenergic/BNIP3L), and miR-378 (Casp3) consistently demonstrate cardioprotective and anti-apoptotic effects, making them the most advanced candidates for clinical biomarker panels and therapeutic targeting. Pro-apoptotic signatures such as miR-1, miR-34a, and miR-100, along with exploratory axes including miR-101, miR-199a, miR-145, miR-20a, and miR-92a, provide additional mechanistic depth but necessitate further validation. The immediate translational action is to evaluate multiplex plasma/serum panels (with rigorous pre-analytical control and clinical endpoints) and simultaneously assess drug–miRNA response pairs (e.g., carvedilol–miR-133a; doxorubicin–miR-30) to enable both risk stratification and miRNA-directed interventions.

## 4. MicroRNA Biomarkers in Coronary Artery Disease

Coronary artery disease (CAD) remains a foremost global health challenge, primarily due to their late diagnosis and the limited availability of highly sensitive and specific biomarkers [42]. CAD is a major contributor to illness and death, particularly in older adults. It is a long-lasting, progressive ailment defined by the accumulation of atherosclerotic plaques in the inner lining of the coronary arteries [43,44]. These plaques restrict blood flow to the heart muscle and are typically associated with underlying inflammation. CAD is represented along a continuum of conditions that differ in severity and urgency [45]. At the milder end, one finds stable angina pectoris, which is defined by predictable chest pain that is triggered by physical exertion or stress and is typically relieved by rest or medication [46]. More severe forms are categorized as acute coronary syndromes (ACSs), which include unstable angina (UA)—characterized by chest pain that occurs at rest or with minimal exertion—and MI, commonly known as a heart attack [47]. Current estimates suggest that about 20.5 million people are likely to die from CVDs in 2025 [48]. Since CAD, commonly known as ischemic heart disease, represents roughly half of all deaths linked to CVDs, it can be deduced that close to 10 million deaths in 2025 will stem from complications related to CAD [49,50]. While CAD mortality rates are lower among Asian populations relative to Western societies, there has been a discernible increase in CAD cases in recent years [51,52]. This escalation is predominantly attributed to the growing incidence of dyslipidemia, obesity, type 2 diabetes, hypertension, and an aging populace (Table 2) [53,54].

In patients suffering from CAD, there is a significant reduction in miR-126 levels, while placenta growth factor (PLGF) levels show an increase, especially in cases that are unstable. The negative correlation between miR-126 and PLGF implies that miR-126 may serve a protective role in CAD by modulating vascular inflammation and maintaining plaque stability [55]. Certain microRNAs, particularly miR-208a, which is specifically expressed in heart muscle and is involved in the regulation of myosin heavy chain production during cardiac development, could significantly enhance the diagnostic process for myocardial infarction [88]. The investigation into circulating miRNAS in apoE knockout mice validated their connection to human CAD. Five miRNAS were identified as altered in apoE−/− mice. In particular, miR-34a, miR-21, and miR-23a were significantly raised in the plasma of CAD patients when compared to healthy individuals [61]. miR-133b and miR-21 are considered potential biomarkers for early identification of CAD. In a study involving 147 participants, miR-133b exhibited a significant downregulation (4.6-fold), while miR-21 demonstrated an upregulation of approximately 2-fold in patients with CAD. Both of these miRNAS displayed a robust correlation with the severity of the disease, particularly in cases of acute coronary syndrome (ACS) [56]. The role of plasmamiR-503 in coronary collateral circulation (CCC) was investigated in patients with coronary artery disease. The researchers found that lower levels of miR-503 were linked to improved CCC formation and higher levels of VEGF-A, a vital factor in angiogenesis. miR-503 was negatively correlated with both VEGF-A and the CCC grade [59].

In a cohort of 255 hyperlipidemia patients compared to 100 healthy controls, significant elevations in plasma levels of miR-122 and miR-370 were observed among the patients. These miRNAS showed a positive correlation with cholesterol, triglycerides, and LDL-C levels. Additionally, higher concentrations of these miRNAS were independently associated with the presence and severity of CAD, as determined by the Gensini score [65]. This research assessed the diagnostic capabilities of circulating miR-208a and miR-370 in the context of CAD. The plasma concentrations of these two miRNAS were found to be significantly elevated in CAD patients when compared to healthy controls [57]. Profiling of serum miR demonstrated that miR-126 was markedly upregulated in individuals suffering from CAD. In a mouse model designed to study atherosclerosis, the overexpression of miR-126 was associated with a reduction in the formation of atherosclerotic plaques and inflammation, while its suppression produced the opposite outcome [60]. Recent evidence highlights the importance of lncRNA–miRNA–mRNA regulatory networks in atherosclerosis progression. Analysis of carotid plaque RNA profiles has identified lncRNA FGF7-5 and lncRNA GLRX3 as protective regulators acting through the miR-2681-5p/ERCC4 axis. Both lncRNAs function as competing endogenous RNAs, suppressing miR-2681-5p and thereby restoring ERCC4 expression, a hub gene involved in DNA repair and cell survival. Experimental models show that loss of FGF7-5 and GLRX3 enhances ox-LDL–induced endothelial apoptosis and TP53 activation, whereas ERCC4 overexpression counteracts these effects [89].

This study explores the role of miR-146a/b and TLR4 signaling pathway in CAD, in addition to the effects of combined statin and renin–angiotensin system inhibition therapy. Elevated levels of miR-146a/b and TLR4-related molecules (IRAK1, TRAF6, TLR4) were significantly observed in CAD patients when compared to non-CAD controls, with a positive correlation between these factors. After 12 months of combined therapy with atorvastatin and either telmisartan (ARB) or enalapril (ACEI), a significant reduction in expression levels was recorded, particularly in the ARB group [73]. The expression of circulating miR in patients with CAD is influenced by maximal exercise (ergospirometry). Blood samples from 20 CAD patients (10 males and 10 females) were analyzed before and after the exercise regimen. Out of 187 miRNAS evaluated, 33 exhibited significant changes following exercise, with 16 showing differences that are specific to gender. Nine miRNAS displayed significantly different responses between male and female patients, and these miRNAS are linked to pathways such as glucose metabolism, oxidative stress, and angiogenesis [77]. This research assessed the diagnostic significance of Nourin-dependent microRNAs, specifically miR-137 and miR-106b-5p, as early indicators of myocardial ischemia in individuals suspected of having CAD. In a cohort of 70 participants, which included patients undergoing stress tests, those with ST-elevation myocardial infarction (STEMI), and healthy controls, both miRNAS exhibited a notable upregulation in CAD patients who had positive results from stress tests. The levels of miR-137 and miR-106b-5p demonstrated a strong correlation with the outcomes of echocardiography and ECG stress tests [72]. Among the 2135 identified miRNAS, 159 were significantly dysregulated—comprising 119 that were upregulated and 40 that were downregulated—in a cohort of 40 CAD patients as opposed to 10 control subjects. The pathways that were most enriched in association with these miRNAS included endocytosis, focal adhesion, and axon guidance [75]. Exosomes from mesenchymal stem cells (MSC-EXOs) are emerging as potential therapeutic agents in the treatment of myocardial ischemia/reperfusion injury (MIRI). This study demonstrates that MSC-EXOs confer protection to cardiomyocytes in the early phases of MIRI by transferring miR-132-3p, which suppresses PTEN, activates the AKT signaling pathway, and enhances insulin signaling. The downstream effects encompass enhanced GLUT4 translocation, increased glucose uptake, and elevated ATP production, thus restoring energy metabolism and reducing myocardial damage [90].

miRNAS are increasingly recognized for their significant potential as non-invasive biomarkers and prospective therapeutic targets in CAD. The heart-specific expression of miR-208a makes it an outstanding diagnostic marker for MI, potentially complementing or even surpassing troponins in the early detection process [88]. The relationship between miR-21 and miR-133b with the severity of ACS indicates their potential utility in risk stratification [57]. In terms of therapy, miR-126’s function in reducing vascular inflammation positions it as a promising candidate for anti-inflammatory treatments, while the modulation of miR-146a through the combined use of statin and renin–angiotensin system inhibitors (e.g., atorvastatin and telmisartan) points to a possible synergistic interaction with existing therapies [69]. The findings concerning miRNA biomarkers in CAD are hindered by methodological issues. A number of studies, particularly those examining miR-126 and miR-146a, are based on limited cohorts (e.g., 147–255 patients) or animal models (e.g., apoE knockout mice), which diminishes the generalizability of the results [55,56,57,67]. Inconsistent outcomes, such as the protective role attributed to miR-126 in some research versus its variable expression in other investigations, may be due to differences in patient demographics, stages of the disease, or the detection methodologies used [55,65].

An expanding collection of studies emphasizes circulating miRNAs as potential diagnostic and prognostic biomarkers for CAD. This review incorporates over 30 investigations, featuring patient groups that vary from small pilot studies (*n* < 30) to extensive clinical populations (*n* > 500), alongside both in vitro and in vivo research. In particular, miR-21, miR-146a, miR-133a/b, miR-126, and miR-145 have been frequently identified in clinical research, exhibiting consistent patterns of upregulation or downregulation linked to disease severity, plaque stability, or myocardial damage. ROC curve analyses across various studies indicated moderate to high diagnostic accuracy (AUCs often exceeding 0.75), particularly in differentiating STEMI, stable angina, or the severity of CAD (e.g., SYNTAX or Rentrop grading). Numerous studies have also validated miRNA–target gene interactions (e.g., miR-126 → S1PR2, miR-21 → LRP6, miR-96-5p → BCL2L13) or pathway influences on inflammation, apoptosis, and lipid metabolism. Additionally, some combined omics or computational methodologies (e.g., SVD, ARM) have further substantiated their relevance to specific diseases. In spite of the increasing evidence, only a small fraction of studies have progressed to translational phases beyond biomarker discovery (T1). Many remain in the observational (T0) or preclinical (in vivo/in vitro) phases. Future investigations should prioritize validation in multicenter prospective cohorts, standardization of assays, and integration with imaging or clinical risk assessments to facilitate the transition of these candidates into clinical practice.

## 5. MicroRNA-Mediated Regulation in Cardiac Hypertrophy

Cardiac hypertrophy is initially an adaptive response to pathological stimuli that helps maintain cardiac function; however, if sustained, it can progress to heart failure. Recent investigations underscore the vital role of miRNAS in modulating this process [91,92]. During the early phases of embryonic development, the heart is formed from various precursor cells that evolve into specialized cardiac structures. In adults, cardiomyocytes exhibit a response to stress through hypertrophy, which, if sustained, can ultimately lead to heart failure [5]. Cardiac hypertrophy, an increase in the size of cardiomyocytes to ensure adequate cardiac output, can be induced by biomechanical stress and pathological stimuli. Although this response is initially adaptive, chronic hypertrophy can lead to heart failure [93]. When faced with stress or injury, an adult heart exhibits hypertrophy and remodeling to sustain its function; nevertheless, persistent changes can often culminate in heart failure. Investigations reveal that miRNAS are essential in this phenomenon, with various miRNAS being either upregulated or downregulated throughout cardiac hypertrophy (Table 3) [94]. 

NFATc3 facilitates upregulation of myocardin expression; in contrast, miR-9 acts to reduce myocardin expression, thereby modulating cardiac hypertrophy [95]. LPA1 and LPA3 exhibit opposing subtype-specific functions in the mediation of cardiomyocyte hypertrophy, with LPA1 identified as a target of miR-23a. This finding reveals a relationship between miR-23a and LPA receptor signaling in the process of cardiomyocyte hypertrophy [96]. The targeted inhibition of miR-199b using a specific antagomir normalized the expression of Dyrk1a, reduced activity of nuclear NFAT, and resulted in a notable inhibition and even reversal of hypertrophy and fibrosis in mouse models of heart failure [97].

**Table 3:** Key microRNAs involved in cardiac hypertrophy and remodeling: experimental models, mechanisms, and targets.

Organism	miRNA and/or Targets	Reference	Organism	miRNA and/or Targets	Reference	Organism	miRNA and/or Targets	Reference
Rat	miR-101 **↓→** Rab1a **↓→** fetal gene expression, protein synthesis, and cell size **↓**	[98]	Mouse and Human	miR-133 **↓→** RhoA, Cdc42, Nelf-A **↑→** cardiac hypertrophy **↑**	[99]	Human and Mouse	miR-499 **↑→** target mRNAs **↓** (incl. Akt, MAPKs). **-**	[100]
Rat and Mouse	miR-145 **↑→** GATA6 **↓→** ANF, BNP, β-MHC **↓**; ERK1/2, JNK, Akt-GSK3β **↓**	[101]	Mouse	miR-155 **↑→** TP53INP1 **↓→** fibroblast proliferation/myofibroblast transition **↑→** cardiac remodeling **↑**	[102]	Mouse and Human	miR-199b **↑→** Dyrk1a **↓→** NFAT activity **↑→** hypertrophy & fibrosis **↑**	[97]
Rat	High glucose **→** miR-150 **↓→** p300 **↑→** hypertrophy **↑**; PKCβ2 mediates miR-150 downregulation.	[103]	Rat	LPA **→** miR-23a **↑→** LPA1 **↓→**hypertrophy **↑**; LPA3 mediates miR-23a upregulation via PI3K/AKT.	[96]	Mouse	miR-133a **↓** in TAC and ISO; overexpression **→**QT prolongation, reduced fibrosis.	[104]
Mouse and Rat	Isoproterenol/aldosterone **→**miR-9 **↓**, NFATc3 **↑→** myocardin **↑→** cardiac hypertrophy **↑**	[95]	Rat	miR-350 **↑→** MAPK11/14, MAPK8/9 **↓→** p38 & JNK **↓→** NFATc3 **↑→**hypertrophy & apoptosis **↑**	[105]	Human, Mouse and Rat	miR-21 **↑→** Spry1 **↓→** CTGF, lysyl oxidase, Rac1-GTP **↑→** atrial fibrosis **↑**	[106]
Mouse and Rat	miR-328 **↑→** SERCA2a **↓→** intracellular Ca^2+^ **↑→** calcineurin **↑→** NFATc3 **↑→** hypertrophy **↑**;	[107]	Mouse and Human	miR-199b **↑→** Dyrk1a **↓→** NFAT activity **↑→** hypertrophy & fibrosis **↑**	[97]	Mouse	miR-27b **↑→** PPAR-γ **↓→** hypertrophy & dysfunction **↑**; TGF-β1 **↓** miR-27b	[108]

↑ indicates upregulation/activation, ↓ indicates downregulation/inhibition and → indicates a causal or sequential relationship (“leads to” or “results in”).

Reduced expression of miR-133a results in improved myocardial fibrosis and diastolic function, with no effect on the level of hypertrophy [104]. The upregulation of miR-21 expression was positively associated with collagen content in the atria and correlated with a reduction in Spry1 protein expression, while simultaneously showing increased levels of connective tissue growth factor (CTGF), lysyl oxidase, and Rac1-GTPase [106]. In a mouse model of heart failure caused by pressure overload, the silencing of miR-27b through a targeted antagomir led to elevated levels of cardiac PPAR-γ expression and diminished cardiac hypertrophy and dysfunction [108].

miRNAS are emerging as promising biomarkers and therapeutic targets in cardiac hypertrophy. The majority of research conducted is preclinical, relying on rodent models of pressure overload, neurohumoral influences (such as isoproterenol and aldosterone), or high-glucose environments, alongside neonatal cardiomyocytes, often with small sample sizes. Only a limited number of studies incorporate human cardiac tissue—specifically miR-21 (associated with atrial fibrillation in atria), miR-199b (linked to failing ventricles), miR-499 (related to failing or hypertrophied hearts), and some data on miR-133a—indicating these possess the most significant translational potential. In terms of biomarkers, miR-21 and miR-199b (which are upregulated in disease states) and miR-499 (elevated in cardiomyopathy) show the greatest promise; miR-133a is indicative of structural and electrical remodeling and may assist in risk assessment. Regarding therapeutic approaches, animal studies demonstrate that anti-miR strategies—particularly anti-miR-21, anti-miR-199b, and anti-miR-328—can reverse conditions, while other candidates (miR-101, -145, -150, -9, -155, -23a, -350 and -27b) are still in preliminary stages, supported only by cellular and animal evidence. To advance towards clinical application, the field requires larger cohorts of human subjects (ideally focusing on circulating miRNAs), longitudinal prognostic evaluations, and comprehensive delivery and safety protocols for leading candidates. In summary: the prospects are promising yet still in the early stages; miR-21 and miR-199b are the most advanced towards translation; miR-499 shows a strong correlation with human conditions; and other candidates are mechanistic leads pending human validation.

## 6. MicroRNA Contributions to the Molecular Basis of Cardiac Arrhythmias

Cardiac arrhythmias are customarily categorized according to their site of origin, which can be either supraventricular or ventricular [109]. In addition to their anatomical source, they are also classified by heart rate, being either bradyarrhythmias or tachyarrhythmias [109]. The development of arrhythmias is primarily attributed to three key electrophysiological mechanisms: reentry, increased automaticity, and triggered activity [110]. However, the underlying mechanisms are not fully clarified, which complicates treatment approaches. While antiarrhythmic medications are used to manage symptoms and prevent recurrence, their impact on survival is limited, and the risk of side effects is a matter of concern [111]. Reentry, in particular, is the most commonly identified mechanism and is generally associated with areas exhibiting slowed electrical conduction [112]. This slowing is frequently due to fibrosis, modifications in ion channel activity, or disrupted intercellular electrical coupling. Sodium channels (I_Na_) are essential for intracellular signaling in cardiac conduction, while connexin-43 (Cx43) facilitates cell-to-cell transmission [113]. When these components malfunction, conduction may be slowed, leading to unidirectional block and reentry, which can cause arrhythmias [112]. Atrial fibrillation (AF) is the arrhythmia most often seen in clinical practice and has a considerable effect on both morbidity and mortality. AF is estimated to affect around 1–2% of the general population, with its prevalence increasing to 8–10% in people older than 80 [114,115]. Although animal models are beneficial, they possess considerable limitations in the study of AF. Clinical AF is a multifaceted disorder that results from numerous pathological processes, often entailing multiple risk factors in one patient [116,117]. AF stands as the most common arrhythmia in North America and Western Europe, with its prevalence growing as populations age [118]. Correspondingly, there is an increase in AF cases in developing countries, which is resulting in a heightened socioeconomic burden [119]. In heart failure (HF), arrhythmias frequently occur due to impaired conduction resulting from factors like ischemia, fibrosis, or genetic elements. Since transcriptional changes are crucial to the progression of HF, it is imperative to understand the molecular basis [120]. The involvement of miRNAS is pivotal in the onset of AF through their regulation of atrial remodeling processes. The expression of these miRNAS, which can be either increased or decreased, is genetically influenced and impacts the progression of the disease. Changes in miR levels in blood and cardiac tissue are linked to cardiovascular issues, including AF, due to their effect on myocardial remodeling (Table 4) [121,122].

The overexpression of miR-1 is linked to the onset of cardiac arrhythmia due to its interference with intracellular trafficking, particularly in the context of calcium handling. It directly targets Stx6, leading to its downregulation, which impairs calcium channel operation and causes an elevation in intracellular calcium levels [123]. Tanshinone IIA is effective in preventing sudden cardiac death by lessening ischemia-induced arrhythmias. It operates through the suppression of miR-1 and restoration of Kir2.1/IK1 potassium channel function [126,144]. miR-365 significantly influences regulation of human cardiac action potential duration by targeting important repolarizing ion channels, notably impacting I_ks_ current. In patient-specific iPSC-derived cardiomyocytes, increasing miR-365 resulted in a prolonged action potential in Short QT syndrome, while its inhibition normalized extended action potentials associated with Long QT syndrome [134]. A high-fat diet contributes to an increased risk of atrial arrhythmia by reducing the speed of atrial conduction, which is associated with miR-27b-mediated downregulation of Cx40. miR-27b directly targets Cx40, an important gap junction protein, and its inhibition can lead to restoration of Cx40 levels [132]. Elevated miR-130a levels in the heart are responsible for causing atrial and ventricular arrhythmias by directly downregulating Cx43, a key protein in gap junctions. Osbourne et al. demonstrated that overexpression of miR-130a in cardiomyocytes downregulates the gap junction protein Connexin43 (Cx43), leading to cardiac arrhythmias [135].

The expression of miR-328 is increased in AF and it contributes to electrical remodeling by downregulating genes associated with L-type Ca^2+^ channels (CACNA1C and CACNB1). This downregulation leads to a decrease in calcium current and a shortening of action potential duration, which enhances susceptibility to AF. Targeting miR-328 could be a potential therapeutic strategy for managing AF [141]. Chronic atrial fibrillation (CAF) raises miR-21 concentrations in human atrial myocytes, directly suppressing the subunits of L-type calcium channels (CACNA1C and CACNB2). Consequently, this leads to a decrease in ICa,L current, thereby contributing to electrical remodeling and maintenance of atrial fibrillation [127]. The expression of miR-1231 is significantly increased in both human and rat hearts after MI, contributing to arrhythmia by directly inhibiting calcium channel gene CACNA2D2. When miR-1231 is overexpressed, arrhythmias worsen; conversely, its inhibition leads to enhanced electrical stability in hearts suffering from MI. The knockdown of CACNA2D2 produces effects akin to those of miR-1231, thereby establishing it as a functional target [137].

The levels of miR-1 and miR-133a were found to be significantly increased in pediatric patients diagnosed with supraventricular arrhythmias (SVas), whereas levels of miR-133b were diminished in both ventricular (Va) and SVa cases when compared to control subjects. These circulating miRNAS exhibit promise as non-invasive diagnostic biomarkers for differentiating between subtypes of pediatric arrhythmias, particularly miR-133a and miR-133b, as indicated by ROC curve analysis [133]. The expression of miR-483-5p was notably elevated in both atrial tissue and preoperative serum of individuals who later developed postoperative atrial fibrillation (POAF) after undergoing coronary artery bypass graft surgery. Among 16 differentially expressed miRNAS, miR-483-5p demonstrated the most significant serum-based predictive capability, with a ROC analysis accuracy of 78% [142]. The dysfunction of ZFHX3 gene, which is acknowledged as a contributor to AF risk, causes modifications in microRNA expression, notably the downregulation of miR-133a and miR-133b. This change fosters atrial remodeling via heightened adrenergic and Wnt/calcium signaling pathways. The application of miR-133a/b mimics led to a suppression of pro-arrhythmic signaling and a reduction in atrial ectopy in mice [128].

This study uncovers novel miR-17 as a newly upregulated microRNA that exacerbates hypothermic ischemia–reperfusion arrhythmias, a complication associated with cardiothoracic surgeries. miR-17 directly interacts with Gja1, resulting in lowered Cx43 levels, which hinders electrical conduction. It also triggers PKC/c-Jun signaling pathway, which is implicated in myocardial injury and the onset of arrhythmias. By inhibiting miR-17, conduction improved, apoptosis and edema were reduced, and the duration of arrhythmias was shortened [140]. In this multicenter investigation, miR-15a-5p, miR-16-5p, and miR-92a-3p are identified as significantly elevated in patients suffering from arrhythmogenic right ventricular cardiomyopathy (ARVC), particularly in those deemed at greater risk for adverse cardiac events. These circulating miRNAS demonstrated superior performance compared to current diagnostic measures in stratifying patients according to 5-year event-free survival [143].

A variety of microRNAs are emerging as significant biomarkers. MicroRNAs are known to regulate multiple arrhythmogenic pathways by targeting ion channels and gap junction proteins. Numerous investigations have utilized in vivo models involving mice or rats to examine the regulatory effects of miRNAs such as miR-1, miR-130a, miR-206, miR-27b, and novel-miR-17 on arrhythmia through the downregulation of critical proteins including Cx43, Kir2.1, Gja1, and CACNA2D2. In various instances, the inhibition of these miRNAs led to a decrease in the severity of arrhythmia, indicating their potential as therapeutic targets for anti-miRNA or miRNA-mimic strategies. Human studies typically encompassed larger cohorts—such as 112 patients diagnosed with AF, 125 with LQTS, 102 with ARVC, and 485 suffering from chronic heart failure. In these investigations, circulating miRNAs like miR-15a-5p, miR-16-5p, miR-92a-3p, miR-155, and miR-483-5p were significantly altered, demonstrating promising diagnostic and prognostic capabilities. For instance, several of these miRNAs achieved ROC AUC values exceeding 0.75 for the identification of high-risk patients, particularly in cases of ARVC and postoperative AF. Crucially, advanced translational models—including patient-derived iPSC cardiomyocytes, atrial biopsies, and ex vivo myocardial tissue slices—have shown that miRNAs such as miR-365, miR-328, and miR-21 influence action potential duration and electrical remodeling by targeting ion channels associated with repolarization (e.g., CACNA1C, CACNB2). In conclusion, these studies illustrate that miRNAS are integral not only to the process of arrhythmogenesis but also serve as promising instruments for clinical risk stratification, diagnosis, and therapeutic innovation. While certain miRNAs are approaching readiness for clinical biomarker application, others present novel intervention strategies through gene- or oligonucleotide-based therapies.

## 7. Prognostic Relevance of MicroRNA Expression in Heart Failure

HF constitutes a major public health burden, primarily due to its high prevalence and dismal prognosis. Heart failure constitutes a leading health concern globally, significantly affecting both illness and death rates [145,146]. Its prevalence is on an upward trajectory, with projections estimating that it could impact up to 10% of the population by 2030 [147]. Large-scale research has identified particular miRNAs that are altered in individuals suffering from HF, highlighting their potential roles in disease progression and their applicability as biomarkers or therapeutic targets [148]. Located within various tissues, they play a significant role in critical disease processes, including cardiac fibrosis and hypertrophy. The role of miRNAs in HF therapy has become increasingly prominent, especially in relation to heart failure with preserved ejection fraction (HFpEF) [149]. HFpEF introduces a unique challenge due to its complex underlying mechanisms and the current lack of effective targeted therapies. In response to various pathological conditions, including prolonged pressure overload, ischemic damage, and neurohormonal disturbances, cardiomyocytes undergo hypertrophy [150,151]. Initially, this enlargement functions as a compensatory mechanism to preserve cardiac output by boosting the strength and contractility of the heart muscle. However, should stress endure, this adaptive response may become maladaptive, ultimately leading to the death of cardiomyocytes and contributing to the progression of heart failure (Table 5) [152,153].

miR-210, classified as a hypoxia-inducible microRNA, is significantly elevated in plasma, mononuclear cells, and skeletal muscle in both animal models and patients experiencing congestive heart failure (CHF). In human subjects, higher levels of miR-210 were associated with a worse NYHA classification (III–IV), while patients with decreasing BNP levels showed lower plasma miR-210 levels [154]. Distinct microRNAs are notably enriched in cardiac mitochondria during both early and late stages of heart failure in a mouse model. Utilizing small RNA sequencing of isolated mitochondria after transverse aortic constriction (TAC), researchers observed that miR-696, miR-532, miR-690, and miR-345-3p were significantly enriched in the early phase of heart failure [157]. A multi-cohort study reveals that a panel consisting of 8 circulating microRNAs, when used in conjunction with NT-proBNP, significantly enhances diagnosis and classification of nonacute heart failure, particularly in cases of HFpEF [160]. Following MI-induced heart failure, miR-21 shows a significant increase in atrial tissue, contributing to the development of atrial fibrosis and an increased risk of AF. In a rat model, targeted knockdown of miR-21 in the left atrium resulted in decreased fibrosis (collagen I and III), normalization of Sprouty-1 expression (an anti-fibrotic target), and a substantial reduction in the duration of AF [163]. Among frail patients, there was a noted downregulation of miR-126, miR-342-3p, and miR-638, while miR-21 and miR-92 were upregulated. The use of empagliflozin, as opposed to metformin or insulin, mitigated the maladaptive increase in miR-21 and miR-92, suggesting a restoration of endothelial function. These results underscore the role of SGLT2 inhibition as a therapeutic strategy and a modulator of endothelial miRNA signatures in diabetic HFpEF [180].

A total of six miRNAS have been recognized as having increased levels in individuals with heart failure, particularly highlighting miR-423, which exhibits a robust association with the clinical diagnosis of the disease [164]. In a considerable cohort of chronic heart failure patients from GISSI-HF trial, the levels of circulating miR-132 were analyzed for their prognostic relevance. Although elevated miR-132 levels were linked to more severe HF, reduced miR-132 levels independently forecasted a higher risk of HF hospitalization but did not predict mortality [155]. The levels of miR-145 are markedly diminished in individuals suffering from AMI and HF when compared to those without coronary artery disease. A significant correlation exists between reduced miR-145 levels and increased cardiac biomarkers, including B-type natriuretic peptide and troponin T, alongside a decline in ejection fraction [161]. The miR-17–92 cluster, with an emphasis on miR-18a, miR-19a, and miR-19b, is implicated in age-related heart failure. In aging hearts that are at risk of failure, these microRNAs were found to be downregulated, resulting in an upsurge in the expression of extracellular matrix (ECM) proteins CTGF and TSP-1, both of which serve as markers for pathological cardiac remodeling [167]. The Phase 1b clinical trial investigated CDR132L, an antisense therapy aimed at miR-132, in patients with chronic ischemic heart failure. Elevated miR-132 concentrations are known to encourage cardiac remodeling, and CDR132L intends to prevent this occurrence. The findings revealed that CDR132L was safe, well tolerated, and produced a sustained, dose-dependent reduction in miR-132 levels in plasma [156]. Anti-miR-92a agent MRG-110 has finalized a Phase 1 proof-of-mechanism study involving human subjects, which further underscores that clinical development is still in its preliminary stages [181]. In stressful situations like myocardial infarction or angiotensin II stimulation, cardiac fibroblasts release extracellular vesicles (EVs) that are abundant in miR-27a [159]. In a cohort of 139 individuals, 32 miRNAs were analyzed in conjunction with NT-proBNP, troponin I, suPAR, and galectin-3. The application of principal component analysis uncovered distinct miRNA patterns (PCs) that correlated with ventricular size and ejection fraction. Notably, both conventional biomarkers (NT-proBNP, galectin-3, suPAR) and specific miRNA PCs were found to independently forecast adverse outcomes, which included hospitalization, transplantation, LV assist device implantation, or mortality [182].

miR-133a is found to be downregulated in the hearts of both chronic heart failure (CHF) rats and patients. The overexpression or mimicry of miR-133a has been shown to enhance cardiac function and decrease fibrosis, whereas the inhibition of miR-133a led to a deterioration in heart structure and performance. These advantageous effects are associated with the inhibition of Akt signaling, as the application of an Akt inhibitor negated the improvements induced by miR-133a [165]. miR-150-5p exhibited a notable downregulation in patients with acute heart failure (AHF) when compared to healthy control subjects as well as individuals experiencing mild to moderate HF. Its expression levels were found to be associated with maladaptive cardiac remodeling, severity of the disease, and clinical outcomes [168]. The miR-221/222 family plays an essential protective role in preventing myocardial fibrosis associated with heart failure due to pressure overload. Studies conducted on both human and mouse models revealed that reduced levels of miR-221/222 were connected to an increase in cardiac fibrosis and ventricular stiffness. Experimental suppression of these miRNAs resulted in an exacerbation of fibrosis and cardiac dysfunction, while their overexpression effectively inhibited TGF-β-mediated profibrotic signaling in cardiac fibroblasts [172].

Sirt1 has a protective function in heart failure by modulating the NF-κB p65/miR-155/BDNF signaling pathway. In a rat model of heart failure, Sirt1 expression was reduced, while NF-κB p65 and miR-155 were found to be upregulated, and BDNF was downregulated, which contributed to cardiac dysfunction and increased apoptosis. The overexpression of Sirt1 resulted in decreased acetylation of NF-κB p65, suppression of miR-155, restoration of BDNF levels, and improvement in cardiac function [178]. The expression of miR-181c is significantly increased in frail elderly patients who have both diabetes and HFpEF. In vitro experiments have confirmed that miR-181c targets Parkin and SMAD7 in human cardiac fibroblasts, both of which are involved in pathways regulating anti-fibrotic and mitochondrial functions [173]. DNA methyltransferase 1 (DNMT1) plays a harmful role in heart failure by inhibiting mitophagy through the miR-152-3p/ETS1/RhoH signaling axis. In doxorubicin-induced heart failure models, DNMT1 was found to be upregulated, promoting methylation of miR-152-3p promoter and consequently decreasing its expression. This resulted in elevated levels of ETS1 and RhoH, which impaired mitophagy and reduced cell viability [176]. In patients with heart failure with reduced ejection fraction (HFrEF), miR-210-3p, along with NT-proBNP, sST2, and galectin-3, was significantly elevated compared to those with HFpEF [177].

Human studies encompass a spectrum from small discovery groups (approximately 12–50 participants) to extensive cohorts (*n* = 953 and *n* = 1710), as well as a long-term prospective series involving 151 hypertensive individuals with a median follow-up of 8.2 years. Additionally, a cross-sectional analysis was conducted on 270 heart failure patients (HFpEF versus HFrEF). Preclinical investigations include post-myocardial infarction, transverse aortic constriction, isoproterenol, and doxorubicin models in rats and mice, along with studies on cardiac fibroblasts, primary cardiomyocytes, and H9c2 cell lines. Biomarker potential: miR-423-5p demonstrates significant diagnostic efficacy; an 8-miRNA panel enhances accuracy when used in conjunction with NT-proBNP; miR-21 aids in diagnosis and prognosis while predicting the onset of HFpEF in older hypertensive patients; miR-132 sharpens the assessment of rehospitalization risk; miR-145 is diminished in acute myocardial infarction and heart failure, tracking injury; miR-150-5p is reduced in advanced heart failure; and miR-210-3p contributes value within a multimarker approach (alongside NT-proBNP, sST2, and galectin-3). Translational mechanisms and targets: miR-21 fosters fibrosis and atrial fibrillation (with antagonism proving beneficial); miR-30d forecasts response to cardiac resynchronization therapy and exhibits anti-apoptotic properties; restoration of miR-133a enhances cardiac function and diminishes fibrosis through Akt signaling; miR-221/222 mitigates TGF-β-induced fibrosis; EV-miR-27a* induces hypertrophy by inhibiting PDLIM5 (with inhibition being protective); low levels of miR-181b correlate with inflammation; miR-181c targets PRKN and SMAD7 to facilitate fibrosis in diabetic HFpEF; miR-125b-5p is downregulated in dilated cardiomyopathy heart failure, but its overexpression improves cardiac structure and function; the pathway DNMT1 → (↓) miR-152-3p → ETS1/RhoH inhibits mitophagy; and MEG3 → (↓) miR-129-5p → ATG14/Akt connects apoptosis and autophagy to cardiac remodeling. Clinically, circulating signatures assist in diagnosis and phenotyping (miR-423-5p; 8-miRNA+NT-proBNP), prognosis and readmission risk (miR-132), prediction of therapy response (miR-30d for CRT), and therapeutic targeting (anti-miR-132 shows early safety and biological activity in Phase 1b; experimental anti-miR-21, miR-133a replacement, and modulation of epigenetic and lncRNA pathways show potential). In summary, human data validate the utility of biomarkers, while animal and cellular studies delineate actionable pathways.

## 8. MicroRNA Profiles and Fibrotic Pathways in Valvular Heart Disease

VHD is a notable factor in cardiovascular fatalities. Current data reveal that 2.7% of the U.S. population experiences some type of VHD, comprising 0.4% with aortic stenosis, 0.5% with aortic regurgitation, 0.1% with mitral stenosis, and 1.7% with mitral regurgitation [183,184]. The incidence escalates with age, reaching nearly 13% among those aged 75 years and older. Throughout the progression of VHD, the myocardium undergoes sophisticated changes in gene and protein expression that intensify cardiac pathology [185]. The aortic valve, characterized by three layered leaflets and maintained by valve interstitial and endothelial cells, opens and closes in excess of three billion times during a lifetime to oversee blood flow [186]. Cardiomyocytes possess a high density of mitochondria, which dynamically perform fusion and fission (MFF) to ensure cellular vitality. Plasma miR levels have been studied as potential markers for degenerative mitral valve (MV) disease; nevertheless, their clinical utilization remains constrained among cardiologists (Table 6) [187]. This hesitation is partly attributed to inconclusive evidence from studies on degenerative aortic valve stenosis and mitral valve regurgitation [188]. A vital feature of the progression of calcific aortic valve disease (CAVD) is the disordered collagen fibers found in the fibrosa layer near the aorta, resulting in leaflets that are thickened and stiff, thus impairing their biomechanics [189,190]. Moreover, calcific nodules contribute to the worsening of this condition. Despite its connections to inflammation, lipid accumulation, and calcification, the specific mechanisms that drive CAVD are yet to be clarified, with surgical valve replacement being the only available treatment for severe instances [191]. A particular pattern of miR expression, termed a miR signature, has been connected to atrial fibrillation associated with valvular heart disease (AF-VHD). Rather than acting alone, miRNAS engage in complex networks that are shaped by genetic and environmental elements, driving the progression of the disease [192].

Calcification and stenosis of the aortic valve are critical health challenges, identified as the third leading cause of heart disease among adults and the predominant acquired valvular condition in developed countries [184,213]. The presence of a bicuspid aortic valve (BAV), which is observed in 1–2% of the population, serves as the primary risk factor for the development of calcific aortic stenosis [214,215]. BAV is recognized as the most common congenital heart defect, frequently correlated with dysfunction of the aortic valve and the progressive dilation of the ascending aorta [216]. Such dilation, commonly associated with aortic regurgitation, elevates the risk of aortic dissection and may necessitate surgical intervention for prevention [217]. BAV can often be asymptomatic; however, it is commonly linked to critical complications such as aortic stenosis, regurgitation, and dilation of the ascending aorta, which markedly raises the likelihood of dissection and rupture. Innovations in genetics, including research that utilizes family linkage, animal models, and transcriptomic analyses, have substantially improved our understanding of the molecular pathways involved in both valve development and disease [214,218].

The function of microRNAs in modulating mitochondrial apoptosis within VHD was examined. The study revealed an elevation in the expression of pro-apoptotic genes (PUMA, DRP1) alongside a reduction in anti-apoptotic gene levels (ARC, GATA-4). Furthermore, there was a downregulation of miR-15a and miR-29a, whereas miR-214 exhibited an upregulation [195]. This analysis explored the presence of miR-222 in patients suffering from degenerative valvular heart disease (DVHD), particularly among those with AF. It was found that serum levels of miR-222, along with inflammatory markers such as IL-6, hs-CRP, and NT-proBNP, were significantly elevated in DVHD patients with AF in contrast to those without AF [198]. The study focused on the expression of microRNAs in myxomatous mitral valve prolapse (MMVP) and fibroelastic deficiency (FED), the two predominant forms of degenerative mitral valve disease (DMVD). Researchers, using valve tissues that were surgically excised, identified a group of microRNAs that were differentially expressed, including miR-500, miR-17, and miR-203, which distinctly separated MMVP from FED. These microRNAs were found to be linked to genes that are involved in the regulation of extracellular matrix and cellular structure, such as decorin (DCN), ACTA2, and PECAM1 [193]. miR-30b inhibits osteogenic differentiation and apoptosis of aortic VICs by directly targeting essential genes such as Runx2, Smad1, and Caspase-3. The overexpression of miR-30b led to a decrease in markers associated with calcification and cell death, underscoring its protective function [193]. A unique plasma miR signature has been recognized that can differentiate patients with mitral valve prolapse (MVP) and severe mitral regurgitation from healthy individuals. It was found that nine miRNAS were significantly deregulated—five were upregulated (for example, miR-140-3p and miR-150-5p) and four were downregulated (for instance, miR-223-3p and miR-361-5p) [199].

This research focused on plasma miR-21 and global longitudinal strain (GLS) as potential biomarkers for detection of myocardial fibrosis (MF) in patients diagnosed with severe aortic valve stenosis (AVS) while maintaining preserved ejection fraction. The results revealed that miR-21 concentrations were significantly higher in fibrotic myocardial tissue and were linked to reduced GLS and other strain-related parameters [194]. CAVD is characterized by the downregulation of miR-195 and the upregulation of VWF. By directly targeting and suppressing VWF, miR-195 inhibits the p38-MAPK signaling pathway, which in turn diminishes valve calcification [204]. The presence of miR-204 is diminished in calcified human aortic valves and aortic valve interstitial cells (AVICs). The loss of miR-204 encourages osteogenic activity by raising the expression of Runx2 and Osx, resulting in calcium deposition [207]. Extracellular vesicles derived from endothelial cells, which are rich in circ_0008362, contribute to diabetic arterial calcification through the modulation of the circ_0008362/miR-1251-5p/Runx2 signaling axis. Research models demonstrated that these vesicles promote the calcification of vascular smooth muscle cells, while clinical observations connected higher plasma concentrations of circ_0008362 to increased arterial calcification in individuals with diabetes [219].

miR-22 is significantly upregulated in calcified aortic valve tissues and plays a role in promoting osteogenic differentiation of VICs. By targeting CAB39 and inhibiting its function, miR-22 enhances calcification and disrupts the CAB39–LKB1–AMPK–mTOR signaling pathway [205]. The expression of miR-125b is increased in CAVD and is predicted to interact with pro-inflammatory chemokine CCL4, which has also been identified as elevated in diseased valves. CCL4 was primarily associated with macrophages and miR-125b has been shown to regulate its expression in vitro [211]. This study establishes miR-141 as a novel antagonist of BMP-2-mediated calcification in aortic valve disease. In BAV leaflets, miR-141 was found to be significantly downregulated, resulting in amplified BMP-2 signaling and calcification [206]. In patients with BAV undergoing surgical procedures for aortic stenosis, miRNAS have been linked to dilation of the ascending aorta (AD). The study found that miR-let-7e-5p and miR-196a-5p were upregulated and correlated with an increased risk of AD, while miR-17-5p was downregulated. These specific miR expression patterns could be significant in the context of valvular dysfunction and dilation [209].

The modulation of miRNA could potentially disrupt fibrotic pathways; for example, miR-139-5p mimics may inhibit the Wnt/β-catenin pathway, leading to a reduction in valve calcification and fibrosis [199]. Additionally, the overexpression of miR-195 might suppress p38-MAPK, which would prevent ECM stiffening in cases of CAVD [203]. Antagomirs designed for miR-22 could restore AMPK–mTOR signaling, thereby halting osteogenic fibrosis [204]. In the case of BAV, restoring miR-17-5p could help mitigate aortic dilation by targeting fibrotic genes [208]. However, there are translational barriers, including challenges in delivering miRNA to fibrotic tissues, off-target effects on non-cardiac ECM, and the high costs associated with antagomir development. In more than 20 studies, the predominant data originates from human samples, specifically plasma, serum, and surgical valve tissue, with additional support from animal studies and in vitro hAVIC/VIC models to validate underlying mechanisms. The cohorts range from small pilot studies to moderate clinical groups in older demographics, including conditions like aortic stenosis and degenerative valve disease, and encompass phenotype comparisons such as BAV versus TAV and MVP versus FED. The potential of circulating biomarkers is robust: multi-miRNA signatures can accurately distinguish MVP from controls, miR-143-3p and miR-452-5p enhance the diagnosis of aortic stenosis in older adults, miR-222 correlates with atrial fibrillation in degenerative valve disease, and miR-122-5p is associated with poorer recovery of left ventricular ejection fraction after TAVR. Tissue and cell studies consistently identify osteogenic and calcific pathways—miR-30b, miR-93-5p, miR-374a-5p, miR-139-5p, and miR-204 generally inhibit calcification through BMP2/Smad/Runx2, Wnt/β-catenin, or p38-MAPK pathways, while miR-22, reduced miR-141, and reduced miR-195 are linked to the promotion of calcific pathways. The combined application of human samples, in vitro systems, and animal studies bolsters translational confidence: blood-based panels seem viable for noninvasive early or auxiliary diagnosis, especially when integrated with echocardiographic metrics. Prognostically, specific circulating miRNAs, such as miR-122-5p, are associated with remodeling and functional outcomes. Therapeutically, the modulation of target miRNAs through antagomiR or mimic strategies, such as restoring miR-204 or inhibiting miR-22 and miR-141 pathways, provides a rational approach to counteract valve calcification, pending larger, standardized clinical validations.

## 9. MicroRNA–Gene Interactions in Cardiomyopathies

Cardiomyopathy can be genetically inherited due to mutations or acquired from various conditions such as coronary artery disease, hypertension, diabetes, infections, or toxicity from drugs [220]. It is divided into four principal types: dilated, hypertrophic, restrictive, and arrhythmogenic right ventricular cardiomyopathy [221]. Hypertrophic cardiomyopathy (HCM) is a genetic heart disorder characterized by autosomal dominant inheritance, primarily caused by mutations in sarcomeric and Z-disk genes [222]. Recent transcriptomic data alongside high-throughput sequencing suggest that HCM may be linked to complex, polygenic, or non-genetic processes, which include disrupted gene regulation [223]. It affects around 1 in 200 to 1 in 500 individuals and is a leading cause of sudden cardiac death in young individuals and athletes (Table 7) [224,225].

Diabetes is a critical health issue worldwide, with cardiovascular disease accounting for more than half of deaths associated with diabetes [54,238]. It serves as a major cause of heart failure, with its prevalence fluctuating from 1:250 to 1:2500 [239]. In dilated cardiomyopathy (DCM), inflammation plays a dual role, assisting in recovery after injury while also causing further damage when it is excessive or prolonged [240]. Cardiac magnetic resonance imaging delivers intricate structural information, but it faces practical challenges. Biomarkers commonly used, including troponins, NT-proBNP, and C-reactive protein, yield some diagnostic insights, although they are not specific to diseases [241,242]. While commonly referred to as idiopathic, up to 50% of these cases could have a genetic origin, with over 400 pathogenic variants discovered in nearly 60 genes—particularly LMNA and BAG3, which are correlated with adverse outcomes [243,244]. The most common causes of myocarditis and inflammatory cardiomyopathy include viral infections, autoimmune responses, and exposure to toxins [245]. Arrhythmogenic right ventricular cardiomyopathy (ARVC) is a genetic heart disorder distinguished by the presence of fibrofatty replacement in the myocardium, making early diagnosis challenging [246].

The Wnt/β-catenin pathway is essential for the regulation of muscle versus fat cell development. In ARVC, diminished expression of desmoplakin (DSP) causes the release of plakoglobin (PG), which migrates to the nucleus and competes with β-catenin [247]. This interaction suppresses Wnt signaling and encourages adipogenesis via transcription factors including PPARγ and C/EBPα [248]. Arrhythmogenic cardiomyopathy (ACM) is a heart disease that is genetically heterogeneous, with a prevalence estimated to be between 1 in 100 and 1 in 5000 [249,250]. Restrictive cardiomyopathy (RCM) is an infrequent but severe heart ailment, generally caused by infiltrative conditions like amyloidosis or, on occasion, genetic mutations [251].

The presence of miR-21 is more pronounced in cardiac fibroblasts than in cardiomyocytes, and its levels rise during the process of cardiac remodeling in patients diagnosed with aortic stenosis. In a study involving 21 distinct miRNAS identified in the plasma of individuals with hypertrophic cardiomyopathy, it was found that only miR-29a exhibited a correlation with the levels of cardiac fibrosis as determined by MRI [227]. As a significant mediator of peripartum cardiomyopathy (PPCM), miR-146a plays a vital role in this severe heart condition related to pregnancy. The 16-kDa prolactin fragment (PRL) enhances miR-146a in endothelial cells, leading to suppression of NRAS and hindering angiogenesis. miR-146a is then transferred to cardiomyocytes via exosomes, which diminishes the expression of Erbb4, Notch1, and Irak1, contributing to cardiac dysfunction [229]. The circulating miR-29a is the sole microRNA that shows a significant association with both left ventricular hypertrophy and fibrosis in individuals diagnosed with HCM. Out of 21 analyzed miRNAS, 12 exhibited upregulation in HCM; however, only miR-199a-5p, miR-27a, and miR-29a demonstrated a correlation with hypertrophy, while only miR-29a was also linked to fibrosis [227].

This study uncovers a unique circulating microRNA signature—including miR-1, miR-16, miR-26a, and miR-133a—that effectively distinguishes Takotsubo cardiomyopathy (TTC) from both acute myocardial infarction (STEMI) and healthy controls. In particular, miR-16 and miR-26a were significantly elevated in TTC, while miR-133a was more prevalent in STEMI. Additionally, observed decrease in miR-125a-5p and increase in endothelin-1 (ET-1) in TTC lend support to the microvascular spasm hypothesis [233]. This study highlights a circulating microRNA signature—miR-142-5p, miR-143-3p, miR-27b-3p, and miR-126-3p—that differentiates children with dilated cardiomyopathy (CDCM) from healthy controls. Notably, a negative correlation was identified between the levels of miR-126-3p and let-7g and left ventricular ejection fraction, indicating their potential as biomarkers for assessing the severity of heart failure in CDCM [228]. A group of circulating microRNAs—including miR-29a-3p, miR-21-5p, miR-18a-5p, and others—that are markedly correlated with diffuse myocardial fibrosis in patients suffering from HCM. Among 14 miRNAS that were upregulated in fibrotic HCM patients (T1 < 470 ms), 11 demonstrated strong inverse correlations with the degree of fibrosis severity [231]. c-Fos has been recognized as a target of miR-101a, with findings showing that the overexpression of miR-101a resulted in decreased levels of both c-Fos protein and mRNA, as well as its downstream protein, transforming growth factor-β1. Using siRNA to silence c-Fos produced antifibrotic effects similar to those of miR-101a, whereas the forced expression of c-Fos protein nullified the impact of miR-101a in cardiac fibroblasts [252]. The synthetic precursors of miR-24 can enhance the expression of miR-24, leading to a reduction in fibrosis as well as a decrease in differentiation and migration of cardiac fibroblasts (CFs). TGF-β, known as a pathological mediator in fibrotic diseases, was found to elevate miR-24 levels [253].

Research on microRNA expression profiles in HCM identified a significant upregulation of miR-20a-5p in hypertrophic hearts. It was shown that miR-20a-5p enhances cardiomyocyte hypertrophy by targeting and downregulating MFN2, a gene associated with mitochondrial function [237]. A specific six-miR signature (miR-122-5p, miR-133a-3p, miR-133b, miR-142-3p, miR-182-5p, and miR-183-5p) has been consistently found in both cardiac tissue and blood of individuals with AC. This signature was confirmed in a large cohort of 90 patients, revealing high diagnostic accuracy and ability to differentiate AC patients from controls, family members, and other types of cardiomyopathy, thereby indicating its potential as a non-invasive biomarker for AC diagnosis [254]. In ischemic dilated cardiomyopathy (iDCM), miR-16-5p is found to be upregulated and contributes to the progression of the disease by inducing endoplasmic reticulum stress and apoptosis in cardiac cells. The overexpression of miR-16 has been shown to activate the PERK/CHOP ER stress pathway, resulting in heightened cell death and stimulation of autophagy, which could function as a protective response to sustain cellular homeostasis before apoptosis takes place [255]. The focus of this research is regulation of five microRNAs (miR-1, miR-133a, miR-133b, miR-208a, and miR-208b) in myocardial tissue of patients with chronic Chagas cardiomyopathy, compared to individuals with DCM and healthy control subjects [256].

By employing miR-seq and mRNA-seq techniques, a total of 393 dysregulated miRNAS and 808 inversely expressed target genes were discovered in pediatric dilated cardiomyopathy relative to healthy controls. Functional pathway analysis showed that the upregulated pathways were implicated in stem cell differentiation and cardiac contraction, whereas the downregulated pathways were associated with signal transduction and protein phosphorylation [257]. The role of miRNAS as diagnostic and prognostic biomarkers in HCM is of considerable interest, especially for identification of myocardial fibrosis and early phases of the disease. While a specific circulating miR signature has yet to be confirmed, miR-1, miR-133, and miR-29a have indicated potential in differentiating disease stages and subtypes [258]. The research examines molecular mechanisms of DCM by comparing volume overload-induced cardiomyopathy (VCM) and ischemic cardiomyopathy (ICM) through miR-seq and mRNA-seq analysis of myocardial biopsies. It identified 112 mRNAs that were differentially expressed and five dysregulated miRNAS, with pathway enrichment indicating ECM remodeling and mitochondrial activity in VCM, while immune signaling was noted in ICM. Four pairs of negatively correlated miR–mRNA (for example, miR-218-5p–SEMA4A, miR-494-3p–SGMS2) were validated, revealing etiology-specific regulatory networks in DCM [236]. The use of human umbilical cord-derived mesenchymal stromal cells (hUC-MSCs) has been demonstrated to enhance cardiac function and decrease myocardial fibrosis in DCM in mice with both short- and long-term diabetes. Treatment with hUC-MSCs restored miR-133a expression and reduced levels of pro-fibrotic markers (e.g., collagen I/III, Smad3/4) and inflammatory cytokines (IL-6, TNF, IL-1β) [259].

The challenges faced include the delivery of miRNA combinations to designated cardiac networks, the control of off-target effects, and the significant costs involved. Human tissue and blood investigations (e.g., DCM biopsies *n* = 82/21 controls; HCM plasma *n* = 41/41; TTC *n* = 36/STEMI *n* = 27/healthy *n* = 28; pediatric DCM miRNA-seq *n* = 20 DCM/10 controls with validations; HCM diffuse fibrosis *n* = 55/4 controls; MYBPC3-HCM tissue *n* = 6/6; enteroviral cardiomyopathy biopsies; AC cohort discovery tissue/blood plus *n* = 90 validation; iDCM plasma *n* = 168 with subgroups) are integrated with animal and cellular models (STZ rat/mouse, anthracycline rabbit; H9C2, endothelial/cardiomyocyte cultures). In HCM, circulating miR-29a is found to be increased and is associated with both hypertrophy and fibrosis; an 8-miRNA panel linked to T1 mapping identifies diffuse fibrosis (AUC≈0.87). In DCM, cardiac miR-208 is found to be elevated and predicts poorer outcomes; pediatric DCM sequencing identifies 393 dysregulated miRNAs with 808 inversely correlated targets. In enteroviral disease, an 8-miRNA signature predicts viral persistence (AUC≈0.90). In TTC, miR-16/26a/let-7f are elevated, while STEMI shows an increase in miR-1/133a; a 4-miRNA signature effectively differentiates TTC from STEMI (AUC = 0.881). Mechanistically: miR-499 (human/mouse) can aggravate cardiomyopathy; in PPCM, miR-146a is elevated (in plasma/heart) and its inhibition enhances function in mice; MYBPC3-HCM shows an upregulation of miR-204; in HCM tissue and in vitro, miR-20a-5p ↑ → MFN2 ↓ → hypertrophy ↑. In diabetic cardiomyopathy, miR-30d ↑ leads to a decrease in FOXO3a/ARC and an increase in caspase-1/IL-1β/IL-18 (increased pyroptosis); circRNA_012164 ↑ → miR-9-5p ↓ → fibrotic genes ↑. Cellular therapies: hUC-MSCs restore miR-133a, decreasing fibrosis/inflammation (COL1A1/α-SMA/Smad3/4 ↓); MSCs also elevate miR-223-3p, suppressing the NLRP3 axis and pyroptosis. In anthracycline cardiotoxicity (rabbit), early miR-1298-5p and late miR-34a-5p (76-fold) increases correlate with plasma–tissue signals; miR-34a-5p tracks damage. Etiology-specific DCM (volume-overload vs. ischemic) reveals five miRNAs and 112 genes that differ; pairs such as miR-218-5p→(DDX6/TTC39C/SEMA4A)↓ and miR-494-3p→(SGMS2)↓ are distinguishing features. The evidence is limited by small cohort sizes and reliance on animal models, which restricts the generalizability of the findings.

## 10. Developmental MicroRNA Dysregulation in Congenital Heart Diseases

CHDs are defined as structural defects that occur at birth, which include conditions such as ventricular septal defects (VSDs), Tetralogy of Fallot (TOF), atrial septal defects, and coarctation of the aorta [260]. These conditions are the foremost contributors to morbidity and mortality in infants diagnosed with heart issues. CHDs encompass a broad range of structural anomalies, from minor issues like VSDs to more severe malformations such as TOF and hypoplastic left heart syndrome (HLHS) [260]. With an incidence of 4 to 14 per 1000 live births, CHDs are a major cause of mortality among infants. The most frequently encountered cyanotic congenital heart defect is TOF, which affects approximately 5 to 7 out of every 10,000 live births [261,262]. TOF is defined by the obstruction of the right ventricular outflow tract (RVOT) and a substantial ventricular septal defect, often requiring early surgical correction [263]. CHDs occur in roughly 1% of newborns, making them the most common birth anomalies and a leading cause of mortality during prenatal and early postnatal periods. While a portion of these cases is linked to genetic or environmental factors, around 80% are of unknown origin, likely resulting from the complex interactions between genetic and environmental influences [264,265]. VSDs represent 20–40% of all CHD but are responsible for 80% of surgeries associated with CHD. The stability of miRNAS in bodily fluids, along with their regulatory functions, has led to their recognition as potential diagnostic and prognostic biomarkers for CHDs (Table 8) [266,267].

The most common birth defects in humans are congenital heart defects, which can frequently be life-threatening. These conditions typically stem from anomalies in the outflow tract, a structure that develops through coordinated action of cells derived from mesodermal and neural crest origins and is regulated by a variety of transcription factors [286]. Between 1959 and 2009, 61,903 CHD-related deaths occurred, with males making up 55%. Childhood deaths fell from 1460 in 1959 to 154 in 2009. Infant deaths, once over 60% of CHD cases (1959–1963), dropped to 22% by 2004–2008. Mortality rates declined for both sexes, though males remain at higher risk. Since 1989, all age groups have seen reduced mortality, with Poisson regression predicting continued decline [287]. In individuals born with CHD, survival rates for those who receive treatment and those who do not are similar within simple and moderate categories. Roughly 40% of patients in these groups are anticipated to survive without treatment, whereas survival rates with treatment range from 75% to 80% [288].

The plasma samples of patients suffering from VSD reveal an upregulation of miR-498, while simultaneously showing a downregulation of hsa-let-7e-5p, miR-155-5p, miR-222-3p, miR-379-5p, miR-409-3p, miR-433, and miR-487b. The use of miR-1 replacement therapy led to a notable reduction in myocardial fibrosis, better calcium handling, inhibition of apoptosis, and inactivation of mitogen-activated protein kinase signaling pathways. Fibulin-2 (Fbln2), a secreted protein that plays a role in extracellular matrix remodeling, has been identified as a new and authentic target of miR-1 [289]. Lack of miR-208a indicated that this miR is crucial for normal cardiac conduction and for the expression of important cardiac transcription factors, including homeodomain-only protein and GATA4, as well as gap junction protein connexin 40 [290].

miR-143 is necessary for proper morphogenesis of cardiac chambers through its direct repression of adducin 3 (add3), a gene responsible for encoding an F-actin capping protein. The reduction in miR-143 or the disruption of its interaction with add3 results in inhibited F-actin remodeling in ventricular cardiomyocytes, which in turn obstructs their normal growth and elongation, leading to ventricular collapse and a decrease in contractility [291]. Children diagnosed with HLHS present a unique profile of miRNAS, where certain miRNAS, including miR-100, miR-145a, miR-99a, miR-137-3p, and miR-204, are affected by alterations in the right ventricle’s volume loading [292]. In cardiac mesenchymal progenitor cells (CMPCs), the overexpression of miR-155 was found to decrease necrotic cell death by 40 ± 2.3% by targeting receptor interacting protein 1 (RIP1). Additionally, the use of a RIP1-specific inhibitor, Necrostatin-1, or siRNA-mediated knockdown of RIP1 led to reductions in necrosis of 38 ± 2.5% and 33 ± 1.9%, respectively [293]. MicroRNAs from non-cardiomyocyte sources, including miR-29a and miR-29c from fibroblasts and miR-126 from endothelial cells, were expected contributors to the library, given that the distinct cell types within the heart were not isolated [294]. Mice with a deficiency in miR-17∼92 die soon after birth, presenting with lung hypoplasia and a VSD [295].

Certain plasma miRNAS—most notably miR-21-5p, miR-155-5p, miR-221-3p, and miR-26a-5p—demonstrate altered expression patterns in children suffering from CHD as opposed to healthy control subjects. It is particularly noteworthy that miR-21-5p showed a significant rise in cyanotic CHD [271]. The development of CHD is influenced by miR-145, which directly targets and downregulates FXN, a gene essential for mitochondrial function and apoptosis. By impairing the expression of FXN, miR-145 disrupts cellular homeostasis [272]. The identification of maternal serum miRNAS as noninvasive biomarkers is crucial for the prenatal detection of fetal CHDs. Researchers employed SOLiD sequencing followed by qRT-PCR validation to analyze serum samples from pregnant women at 18–22 weeks of gestation. They identified four miRNAS—miR-19b, miR-22, miR-29c, and miR-375—that were significantly upregulated in mothers carrying fetuses with CHD compared to control subjects [275].

In children with congenital heart disease undergoing cardiopulmonary bypass (CPB), remote ischemic preconditioning (RIPC) has been shown to significantly lower the incidence of acute kidney injury (AKI), with rates of 19.0% in the RIPC group compared to 46.2% in the control group. Additionally, RIPC was associated with a significant increase in miR-21 levels in both blood and urine, as well as a notable reduction in inflammatory marker TNF-α [274]. A total of 36 circulating miRNAS that are differentially expressed were identified in patients with VSD when compared to control subjects—15 of which were found to be upregulated and 21 downregulated. Among these, 8 miRNAS were confirmed through RT-PCR, demonstrating consistent expression patterns. Bioinformatic analysis indicated that these miRNAS are likely to regulate genes such as NOTCH1, HAND1, ZFPM2, and GATA3, which play a significant role in cardiac development, especially in morphogenesis of the right ventricle [270].

In cardiac tissue from patients with CHD—including atrial septal defect (ASD), VSD, and TOF—295 dysregulated microRNAs have been identified when compared to non-CHD controls. These altered miRNAS are involved in vital biological processes such as cell proliferation, survival, angiogenesis, migration, and regulation of the cell cycle. Specific miRNAS, such as miR-221-3p, miR-218-5p, and miR-873-5p, have been validated and are associated with roles in cardiogenesis and cardiac function [276]. In umbilical cord blood of fetuses suffering from CHD, the levels of miR-1, miR-208, and miR-499 were found to be significantly diminished in comparison to healthy controls. These miRNAS revealed strong negative correlations with the occurrence of CHD and demonstrated notable predictive accuracy, reflected in AUCs of 0.86, 0.75, and 0.84, respectively [279].

Patients who have undergone repair for TOF display unique circulating miR profiles, with 49 to 77 miRNAS showing significant alterations when compared to healthy controls. Notably, miR-181d-5p, miR-206, and miR-625-5p were confirmed and demonstrated considerable diagnostic potential (AUCs > 0.98) [296]. In infants suffering from TOF, miR-421 is significantly increased in the right ventricular myocardium relative to control groups. There is a strong inverse correlation between miR-421 and SOX4, a vital regulator in Notch signaling pathway that is important for outflow tract development. Experiments focused on functionality have validated that the overexpression of miR-421 diminishes SOX4 levels and may disrupt typical proliferation of cardiac cells [283]. In the right ventricular tissue of 22 non-syndromic TOF patients, dysregulated microRNAs were detected and matched with altered mRNA targets via a stringent prediction pipeline. Key miRNAS, particularly miR-1 and miR-133—vital for cardiac development—were found to be misregulated, influencing target genes like KCNJ2, FBN2, SLC38A3, and TNNI1, all of which are involved in cardiac function [281]. The blood of infants suffering from VSD revealed 22 differentially expressed microRNAs—19 of which were downregulated and 3 upregulated—when compared to healthy controls. These microRNAs are predicted to influence over 10,000 target genes that are involved in cardiac development and morphogenesis. In particular, eight target genes were significantly upregulated in the VSD group and were linked to PKC and PI3K-Akt signaling pathways, both of which are recognized for their roles in heart development [284].

The bulk of evidence is sourced from human data, including plasma or serum, cardiac tissue, and maternal or umbilical-cord blood, and is further substantiated by mouse models (embryonic congenital heart disease [CHD] and right-ventricular pressure loading) and in vitro systems such as H9C2 cardiomyocytes, embryonic endocardial cells, primary right ventricular cells, and human cardiac organoids. Sample sizes vary from small discovery sets with validation to moderate and large clinical cohorts. As for circulating biomarkers, several microRNAs (miRNAs) consistently emerge as significant: miR-21 (increased in CHD-associated pulmonary arterial hypertension [CHD-PAH]; predicts PAH and postoperative critical illness; rises with remote ischemic preconditioning alongside decreased TNF-α and acute kidney injury), miR-8078 (increased with the severity of CHD-PAH; serves as an independent risk factor; enhances diagnostic accuracy when combined with BNP), miR-204 (decreased; negatively correlates with pulmonary pressures), and—after repair of TOF—miR-206, miR-181d, and miR-625-5p (decreased in heart failure, demonstrating strong discrimination). Prenatal signals include maternal serum miR-19b, miR-22, miR-29c, and miR-375 (all elevated; useful as a panel) and umbilical-cord miR-1, miR-208, and miR-499 (all decreased). Mechanistic themes converge on targetable pathways: miR-34a elevation leads to decreased HIF-1α, VEGF, and SIRT1 (resulting in capillary rarefaction; anti-miR-34a treatment restores capillarity and function), miR-145 elevation results in decreased FXN (affecting mitochondria and apoptosis), miR-219-5p elevation leads to decreased LRH-1 and subsequently decreased Wnt/β-catenin (increasing apoptosis; inhibition reverses this effect), miR-592 reduction results in increased KCTD10 and decreased Notch (promoting proliferation and providing protection from a hypoplastic phenotype), miR-34a and miR-421 elevation leads to decreased NOTCH-1 and SOX4, and miR-187 elevation results in decreased NIPBL (affecting endothelial chromatin accessibility and leading to septation defects). From a translational standpoint, the data support (i) non-invasive diagnostics and subtype stratification through circulating miRNA panels (and combinations with BNP), (ii) risk stratification and monitoring (for instance, miR-21, miR-204, miR-206/-181d), and (iii) therapeutic targeting of pathogenic miRNA-target pathways (for example, anti-miR-34a; modulation of Notch/Wnt pathways; miR-187/NIPBL).

## 11. Conclusions

MicroRNAs are diminutive, noncoding RNAs that influence gene expression after transcription and have surfaced as crucial regulators in cardiovascular physiology and pathology. Their resilience in biological fluids and capacity to target numerous genes establish them as both biomarkers and therapeutic targets for a wide range of heart diseases.

In general, miRNAs are not currently prepared for standard clinical application; however, the alignment of experimental findings and preliminary clinical data suggests that certain miRNAs could be incorporated into future cardiovascular practices such as diagnostic instruments, risk assessment tools, or therapeutic objectives. The subsequent phase requires multicenter prospective research, functional validation in human tissues, and the creation of secure, targeted delivery mechanisms to transition the field from mere descriptive profiling to clinically applicable precision medicine. At this time, no miRNA-based therapies targeting cardiovascular disease have reached Phase 3, and no miRNA-based diagnostics have been adopted into everyday cardiology practice or sanctioned by regulatory bodies; recent literature indicates that the evidence for clinical use remains inadequate. Consequently, the practical readiness of miRNA applications for standard care is low, highlighting the urgent need for extensive multicenter trials employing standardized assays and direct comparisons with existing cardiac biomarkers.

Many valuable insights arise from rodent studies; however, the translation of these insights to human subjects is not guaranteed. There are notable differences in cardiac physiology and miRNA expression patterns among species. To counteract this limitation, future investigations should seek to validate their findings in large-animal models or human cardiac cells. The application of patient-derived iPSC cardiomyocytes and multicenter human cohorts is imperative.

In conclusion, miRNAs such as miR-21, miR-133, miR-126, miR-199b, and miR-1 show great potential as diagnostic, prognostic, and therapeutic agents in cardiovascular diseases. Despite compelling preclinical evidence, their clinical application must overcome challenges related to study design, standardization, and delivery. By resolving these issues, microRNAs could significantly impact personalized cardiovascular medicine, leading to improved patient outcomes through targeted diagnostics and therapeutic strategies. miRNAs are potent regulators in cardiovascular biology and hold promise as biomarkers and therapeutics, but challenges remain in clinical translation.

## Figures and Tables

**Table 1:** Experimental studies investigating the roles of microRNAs in cardiomyocyte apoptosis and cardioprotection mechanisms.

Organism	miRNA and/or Targets	Reference	Organism	miRNA and/or Targets	Reference	Organism	miRNA and/or Targets	Reference
Neonatal rat cardiomyocytes	**↑** miR-30b targets **↓** Bcl-2	[20]	Sprague–Dawley rat cardiomyocytes	**↑** miR-15b targets **↓** Bcl-2 affects cytochrome c and caspase-3/9.	[21]	H9c2 rat cardiomyocytes	**↓** miR-101 inhibition leads to **↑** RAB5A **→↑** autophagy **→↓** apoptosis	[22]
Neonatal rat cardiomyocytes	**↑** miR-21 targets **↓** PDCD4	[23]	Rat cardiomyocytes	**↑** miR-133 targets **↓** caspase-9, **↓** caspase-3	[24]	Cardiac myocytes	**↓** miR-199a targets **↑** Hif-1α, **↑** Sirt1	[25]
Mouse cardiomyocytes	**↓** miR-24 in MI (**↑** when treated) targets **↓** Bim	[26]	Rat (in vivo ischemia model and H9c2	**↓** miR-378 in ischemia (**↑** when treated) targets **↓** caspase-3.	[27]	Rats and neonatal rat cardiomyocytes	**↓** miR-1, **↓** miR-133a in IR (**↑** with IPost); miR-133a targets **↓** CASP9	[28]
Cultured cardiac myocytes	**↑** miR-21 targets **↓** PDCD4 **→** affects AP-1 signaling	[29]	Isolated cardiomyocytes and in vivo models (mouse/rat)	**↓** miR-30 targets **↓** β1AR, β2AR, Giα-2, **↓** BNIP3L/NIX	[30]	H9c2 rat cardiomyocytes	**↑** miR-34a targets **↓** Bcl-2	[31]
Rat model and H9C2 cardiomyocytes	**↑** miR-21 targets **↓** PTEN **→↑** Akt **→↑** Bcl-2/Bax **→↓** Caspase-3	[32]	Neonatal mouse cardiomyocytes	**↑** miR-100 targets **↓** IGF1R; **↓** miR-100 **→↑** IGF1R **→↓** apoptosis	[33]	H9C2 rat cardiomyocytes	**↑** miR-1 targets **↓** IGF-1	[34]
Cultured cells	**↓** miR-30 targets **↓** p53 **→↓** Drp1 **→↓** mitochondrial fission	[35]	Rat I/R model and cultured cardiomyocytes	**↑** miR-1 targets **↓** Bcl-2	[36]	Cultured cardiomyocytes	**↑** HIF-1α **→↑** miR-21 (feedback loop); miR-21 modulates **↓** PTEN **→↑** Akt	[37]
Cultured cardiomyocytes	**↑** miR-145 targets **↓** CaMKIIδ	[38]	Neonatal rat cardiomyocytes	**↑** miR-20a (targets **↓** Egln3/PHD3)	[39]	Rat H9c2 cardiomyocytes	**↓** miR-92a (targets **↑** Smad7 **→↓** NF-κB p65 **→↓** apoptosis)	[40]

↑ indicates upregulation/activation, ↓ indicates downregulation/inhibition and → indicates a causal or sequential relationship (“leads to” or “results in”).

**Table 2:** Overview of microRNA expression profiles and their diagnostic/mechanistic roles in CAD.

Organism	miRNA and/or Targets	Reference	Organism	miRNA and/or Targets	Reference	Organism	miRNA and/or Targets	Reference
Human	**↓** miR-126 in CAD patients; **↑** PLGF in UAP and AMI patients.	[55]	Human	**↓** miR-133b and **↑** miR-21 in CAD patients; both correlated with disease severity.	[56]	Human	**↑** miR-208a (*p* = 0.006) and **↑** miR-370 (*p* = 0.003) in patients.	[57]
Human	miR-146a rs2910164 GC and CC genotypes **↑** CAD risk and **↑** mature miRNA expression.	[58]	Human	**↓** miR-503 in both CCC groups vs. controls; lowest in good CCC group; **↑** VEGF-A in good CCC group.	[59]	Human, Mouse (ApoE−/−) and HUVECs	**↑** miR-126 reduced atherosclerotic plaques, **↓** TNF-α, **↓** IL-1β, **↑** IL-10; miR-126 targets S1PR2.	[60]
Mouse (apoE−/−), Human	**↑** miR-34a, miR-21 and miR-23a in CAD patients; differentially expressed miRNAs confirmed in apoE−/− mice.	[61]	Human	**↓** miR-214 in patients; **↑** PLGF in UAP and AMI but not SAP; positive correlation between miR-214 and PLGF in AMI.	[62]	Human	**↓** miR-145, **↓** miR-155, and **↓** let-7c in patients; the combination of three miRNAs provided a diagnostic signature.	[63]
Human	miR-149 rs2292832 and miR-196a2 rs11614913 are significantly associated with CAD.	[64]	Human	**↑** miR-122 and miR-370 in hyperlipidemia; positively correlated with TC, TG, LDL-C and Gensini score.	[65]	Human	**↑** miR-133a-3p, **↑** miR-451a, **↑** miR-584-5p, **↑** miR-21-5p, **↑** miR-221-3p; strongest markers.	[66]
Human	**↑** miR-146a in good CCC, **↓** in poor CCC; VEGF-	[67]	Human, in vitro (macrophage model)	**↓** miR-128-3p in patients; no change in miR-195-5p; miR-128-3p impairs cholesterol efflux in macrophages.	[68]	Human (postmortem)	**↓** miR-126-5p and **↓** miR-499a-5p in CAD-SCD; no change in miR-134-5p.	[69]
Human	**↑** miR-144 in patients; levels correlated with STEMI and higher SYNTAX scores.	[70]	Human	**↑** SGPP1 (target of miR-133b); **↓** ATG5 and **↓** LRP6 (targets of miR-21); expression modulated by miRNA inhibitors.	[71]	Human	**↑** miR-137 (up to 1198-fold) and **↑** miR-106b-5p (up to 122-fold) in CAD patients.	[72]
Human	**↑** miR-146a/b, TLR4, IRAK1, TRAF6 in patients; all decreased after treatment, especially with ARB.	[73]	Human	**↓** miR-96-5p and **↑** BCL2L13 in patients and hypoxic cells;	[74]	Human	159 miRNAs DE (119 **↑**, 40 **↓**); top **↑**: miR-144-3p, miR-34a-5p.	[75]
Human	**↑** miR-27a, **↓** miR-146a; no sig. change in miR-34a and miR-149; correlations with lipid profile and BMI.	[76]	Human	33 miRNAs changed significantly post-exercise; 16 differed by gender; 9 miRNAs showed significant gender-dependent responses.	[77]	Human and cell lines	**↑** miR-6721-5p and **↓** meta-VCL in CAD; miR-6721-5p targets meta-VCL; IL-10 and TNF-α.	[78]
Human	**↓** miR-5187-5p, **↑** miR-199a-5p, miR-182-5p.	[79]	Human	↑ miR-17-5p, ↑ miR-21-5p, ↑ miR-210-3p, ↑ miR-29b-3p, ↑ miR-7-5p and ↑ miR-99a-5p (>2-fold) in patients vs. controls.	[80]	Human	Plasma miR-378 was significantly downregulated in CAD patients and negatively correlated with Gensini score.	[81]
Human	miR-106b-5p expression is significantly elevated in CAD group compared to controls.	[82]	Human	7 miRNAs (miR-10b-5p, miR-29c-3p, miR-142-5p, miR-320b, miR-451a, miR-486-3p, miR-625-3p) differentially expressed.	[83]	Human	Serum miR-299-3p was significantly upregulated in CAD patients compared to controls.	[84]
Human	miRNA146, miRNA21 and MMP1 significantly increased in STEMI vs. SIHD. miRNA126 negatively correlated with LV and arterial function parameters. MMPs correlated with stiffness.	[85]	Human	Higher MMP-9 in oCAD; higher TNF-α in INOCA; miR-145 predictive for INOCA/ANOCA.	[86]	Human	AMPKα1 and let-7g-5p are differentially expressed in EAT vs. VAT; TNFA is upregulated in both tissues in CAD; miR-1247-5p and miR-326 downregulated are in EAT with obesity.	[87]

↑ indicates upregulation/activation, ↓ indicates downregulation/inhibition.

**Table 4:** Key microRNAs involved cardiac arrhythmias: experimental models, mechanisms, and clinical implications.

Organism	miRNA and/or Targets	Reference	Organism	miRNA and/or Targets	Reference	Organism	miRNA and/or Targets	Reference
Mouse	miR-1 **↑**; trafficking-related genes (Stx6, Braf, Ube3a, etc.) **↓**	[123]	Human and Rat	miR-1 **↓**, miR-328 **↓**, miR-664 **↑**, etc.; miR-1 inhibits CACNB2 expression **→↓** Ca^2+^ influx	[124]	Mouse	miR-206 **↑→** Cx43 **↓**; caused arrhythmias, abnormal heart rate and PR interval;	[125]
Rat	Tanshinone IIA **↓** miR-1 **↑** (in MI); restored Kir2.1 and IK1 current;	[126]	Human	CAF **↑** miR-21 **↑→↓** CACNA1C and CACNB2	[127]	Mouse	ZFHX3 KD **→** miR-133a/b **↓**, miR-184 **↑**	[128]
Human	Variants found in miR-1-2 and miR-133a-2 did not affect mature miRNA.	[129]	Human and Mouse	miR-106b-25 cluster **↓→** RyR2 **↑** (42%); **↑** Ca^2+^ spark and SR leak; **↑**	[130]	Mouse	miR-206 inhibitor **↓** arrhythmia severity; **↓** CKMB and cTnI	[131]
Mouse	HFD **↑** miR-27b **↑→** Cx40 **↓**; slowed atrial conduction, increased atrial tachycardia vulnerability	[132]	Human (Pediatric)	SVa: miR-1 **↑**, miR-133a **↑**; Va & SVa: miR-133b **↓** vs. controls; miR-133a higher in SVa vs. Va	[133]	Human	miR-365 **↑→** APD **↑** in Short-QT; miR-365 inhibition **→** APD normalization in Long-QT	[134]
Mouse	miR-130a **↑→** Cx43 **↓** (>90%); caused atrial and ventricular tachyarrhythmias	[135]	Human	AT1R-1166CC genotype **→↑** ICD therapies (ATP + shocks); miR-155 **↓**.	[136]	Human and Rat	miR-1231 **↑** in MI; targets CACNA2D2 **↓**; overexpression **→** arrhythmia **↑**; inhibition **→** arrhythmia **↓**	[137]
Rat	ART **↓** VAS; 16 DEMs altered with ART; MAPK, Wnt, Hippo pathways implicated; rno-miR-370-3p and rno-miR-6319 **↓.**	[138]	Rat and Human	miR-1 **↑** in CAD; **↑** arrhythmias; **↓** KCNJ2 (Kir2.1) and GJA1 (Cx43)	[139]	Rat	novel-miR-17 **↑→** Gja1 **↓→** Cx43 **↓→↓** conduction velocity; activates PKC/c-Jun pathway	[140]
Dog, Human and Mouse	miR-328 **↑** in AF; CACNA1C and CACNB1 **↓**	[141]	Human	miR-483-5p **↑** (atrial and serum); predictive of POAF with 78% accuracy; miR-208a **↓** in POAF tissue	[142]	Human	miR-15a-5p **↑**, miR-16-5p **↑**, miR-92a-3p **↑** in ARVC	[143]

↑ indicates upregulation/activation, ↓ indicates downregulation/inhibition and → indicates a causal or sequential relationship (“leads to” or “results in”).

**Table 5:** Key microRNAs in heart failure: diagnostic, prognostic, and therapeutic implications across experimental models and clinical studies.

Organism	miRNA and/or Targets	Reference	Organism	miRNA and/or Targets	Reference	Organism	miRNA and/or Targets	Reference
Human and Rat	miR-210 ↑ associated with hypoxia/oxygen metabolism	[154]	Human	Higher miR-132 was linked to HF severity, but lower levels predicted HF hospitalizations independently.	[155]	Human	CDR132L caused dose-dependent miR-132 ↓, NT-proBNP ↓	[156]
Mouse	69 miRNAs ↑ and 2 ↓ in early HF; 16 ↑ and 6 ↓ in late HF; miR-696, miR-532, miR-690 and miR-345-3p enriched in early HF mitochondria.	[157]	Human	miR-21 levels (PV and CS) were higher in HF, correlated with EF and BNP; predicted rehospitalization	[158]	Rat	miR-27a ↑ in CHF hearts and EVs; targets PDLIM5 ↓.	[159]
Human	8-miRNA panel effectively identified HF; improved specificity and accuracy when combined with NT-proBNP	[160]	Human	miR-145 ↓ in AMI and HF; negatively correlated with BNP and troponin T; positively with EF	[161]	Rat	miR-21 ↑ in fibroblasts and HFpEF model; upregulates Bcl-2	[162]
Rat	MI rats showed ↑ miR-21, atrial fibrosis, and AF; KD21 reduced miR-21 levels, fibrosis, and AF duration.	[163]	Human	miR423-5p enriched in HF blood; AUC 0.91 (*p* < 0.001); 5 other miRNAs less specific	[164]	Rat	miR-133a ↓ in HF; overexpression improved LV function and reduced fibrosis; Akt inhibition reversed these effects.	[165]
Human and Canine	miR-30d is associated with CRT response, enriched in high-stress myocardium and reduces apoptosis in vitro.	[166]	Mouse and Human	↓ miR-18a, miR-19a/b ↓ linked with ↑ CTGF, TSP-1 in failure-prone hearts; opposite in failure-resistant	[167]	Human	miR-150-5p significantly ↓ in AHF vs. HS and MHF; associated with maladaptive remodeling and disease severity	[168]
Human	miR-155 ↑ is associated with poor cardiac function post-MI.	[169]	Human and Rat	miR-181b ↓ anti-inflammatory; regulates TNF-α, IL-1β, IL-6	[170]	Human	Higher miR-21 levels are significantly associated with HFpEF development.	[171]
Human, Mouse and Rat	miR-221/222 ↓ targets TGF-βR1, TGF-βR2, JNK1 and ETS-1; inhibits SMAD2-mediated signaling	[172]	Human	miR-181c↑ targets PRKN and SMAD7.	[173]	Human and Rat	miR-125b-5p downregulated in DCM-HF	[174]
Rat	miR-132 ↑ targets PTEN, suppresses PI3K/Akt pathway and fibrotic gene expression.	[175]	Rat and Cell Line	miR-152-3p ↓ targets ETS1; ETS1 → RhoH ↑; mitophagy inhibited	[176]	Human	miR-210-3p, NT-proBNP, sST2, and galectin-3 levels were significantly higher in HFrEF than HFpEF.	[177]
Rat	miR-155 ↑ targets BDNF and is regulated by Sirt1/NF-κB p65.	[178]	Mouse	Silencing MEG3 improved cardiac dysfunction, reduced hypertrophy, oxidative stress, apoptosis, autophagy, and fibrosis.	[179]			

↑ indicates upregulation/activation, ↓ indicates downregulation/inhibition and → indicates a causal or sequential relationship (“leads to” or “results in”).

**Table 6:** MicroRNA expression profiles and their functional targets in valvular heart pathologies.

Organism	miRNA and/or Targets	Reference	Organism	miRNA and/or Targets	Reference	Organism	miRNA and/or Targets	Reference
Human	miR-205-3p ↓ → IL-1β ↑; miR-3909 ↓ → IL1R1 ↑	[183]	Human	miR-500, miR-3174, miR-17, etc. ↔ → DCN, ACAN, FMOD, ACTA2, ECM2, DES, ESM1, PECAM1 (in silico predicted; inverse correlations)	[193]	Human	miR-21 ↑ → Myocardial fibrosis ↑; LV strain ↓	[194]
Human	miR-15a ↓ → PUMA ↓; miR-29a ↓ → DRP1 ↓; miR-214 ↑ → ARC ↓	[195]	Human	miR-30b ↓ → Runx2 ↑, Smad1 ↑, caspase-3 ↑	[196]	Canine	miR-30b-5p ↑ in B1 < 3y, B1 3–7y, B1 > 7y vs. A	[197]
Human	miR-222 ↑ → IL-6 ↑, hs-CRP ↑, NT-proBNP ↑	[198]	Human	miR-140-3p ↑, miR-150-5p ↑, miR-210-3p ↑, miR-451a ↑, miR-487a-3p ↑; miR-223-3p ↓, miR-323a-3p ↓, miR-340-5p ↓, miR-361-5p ↓	[199]	Human	miR-139-5p ↓ → RUNX2 ↑, FZD4 ↑, CTNNB1 ↑ → Osteogenesis ↑	[200]
Canine	let-7c ↓, miR-17 ↓, miR-20a ↓ → myofibroblast differentiation/senescence ↑; miR-30d ↓ → apoptosis ↑	[201]	Human	miR-146a ↑ → TLR4/IRAK1	[202]	Human & Mouse	miR-214 ↑ → TWIST1 ↓ → VIC calcification ↑	[203]
Human (in vitro)	miR-195 ↓ → VWF ↑ → p38-MAPK ↑ → ALP/Runx2/OCN ↑ → Calcification ↑	[204]	Human (in vitro)	miR-22 ↑ → CAB39 ↓ → AMPK/mTOR dysregulation → Osteogenesis ↑	[205]	Human & Porcine	miR-141 ↓ → BMP-2 ↑ → ALP ↑ → Calcification ↑	[206]
Human & Mouse	miR-204 ↓ → Runx2 ↑, Osx ↑ → ALP ↑ → Calcification ↑	[207]	Human (in vitro)	miR-629-3p ↓ → TAGLN ↑ → Calcification ↑	[208]	Human	let-7e-5p ↑, miR-196a-5p ↑ → Aortic dilation ↑; miR-17a-5p ↓ (NS)	[209]
Human & Mouse	miR-122-5p ↑ → BCL2 ↓ → Cardiomyocyte viability ↓ → LVEF ↓	[210]	Human	miR-125b ↑ → CCL4 ↓ (targeted in THP-1 macrophages)	[211]	Human (in vitro)	miR-93-5p ↑, miR-374a-5p ↑ → BMP2 ↓ → Smad1/5 ↓ → Runx2 ↓ → Calcification ↓	[212]

↑ indicates upregulation/activation, ↓ indicates downregulation/inhibition and → indicates a causal or sequential relationship (“leads to” or “results in”).

**Table 7:** The Role of microRNAs in the pathogenesis and progression of cardiomyopathies.

Organism	miRNA and/or Targets	Reference	Organism	miRNA and/or Targets	Reference	Organism	miRNA and/or Targets	Reference
Mouse	miR-223-3p ↑ → NLRP3 ↓	[226]	Human	miR-29a → associated with both hypertrophy & fibrosis	[227]	Human	mir-142-5p, ↑ mir-143-3p, ↑ mir-27b-3p, ↑ mir-126-3p, ↑ let-7g ↑ → LVEF ↓	[228]
Human and Mouse	miR-146a ↑ → NRAS, ↓ Erbb4, ↓ Notch1, ↓ Irak1 ↓	[229]	Rat and Cell Line	miR-30d ↑ → foxo3a, ↓ ARC ↓, caspase-1, ↑ IL-1β, ↑ IL-18 ↑	[230]	Human	miR-18a-5p, miR-146a-5p, miR-30d-5p, miR-17-5p, miR-200a-3p, miR-19b-3p, miR-21-5p, miR-193-5p, miR-10b-5p, miR-15a-5p, miR-29a-3p ↑	[231]
Human	miR-208 ↑ → β-MHC, ↑ collagen volume ↑	[232]	Human	miR-16 ↑, miR-26a ↑, let-7f ↑, miR-1 ↑, miR-133a ↑, miR-125a-5p ↓ → ET-1 ↑	[233]	Human	↑ miR-204 → TRPM3 (trend) ↑, troponin I phosphorylation ↓	[234]
Human	miR-135b, ↑ miR-155, ↑ miR-190, ↑ miR-422a, ↑ miR-489, ↑ miR-590, ↑ miR-601, ↑ miR-1290 ↑ → immune gene suppression	[235]	Human	miR-218-5p ↑ → DDX6, ↓ TTC39C, ↓ SEMA4A ↓; miR-494-3p ↑ → SGMS2 ↓	[236]	Human and Cell Line	miR-20a-5p ↑ → MFN2 ↓ → ANP → ↑ hypertrophy ↑	[237]

↑ indicates upregulation/activation, ↓ indicates downregulation/inhibition and → indicates a causal or sequential relationship (“leads to” or “results in”).

**Table 8:** Summary of miRNA dysregulation, targets, and functional roles in congenital heart disease.

Organism	miRNA and/or Targets	Reference	Organism	miRNA and/or Targets	Reference	Organism	miRNA and/or Targets	Reference
Mouse and Human	miR-187 ↑ → NIPBL ↓	[268]	Human	Altered miRNA target sites in HAND1 3′UTR (gain/loss of binding sites)	[269]	Human	let-7e-5p, miR-222-3p, miR-433 → NOTCH1, HAND1, ZFPM2, GATA3	[270]
Human	miR-21-5p ↑, miR-221-3p ↑, miR-26a-5p ↑, miR-15-5p ↑	[271]	Human and cell lines	miR-145 ↑ → FXN ↓	[272]	Mouse (pregnant D7 mice and embryonic endocardial cells)	miR-34a ↑ → NOTCH-1 ↓ → altered Notch signaling	[273]
Human (children with CHD)	miR-21 ↑ → TNF-α ↓	[274]	Human (pregnant women)	miR-19b ↑, miR-22 ↑, miR-29c ↑, miR-375 ↑	[275]	Human	miR-221-3p ↑, miR-218-5p ↑, miR-873-5p ↑ (associated with cardiogenesis and dysfunction)	[276]
Human and rat cardiomyocyte cell line (H9C2)	miR-219-5p ↑ → LRH-1 → ↓ Wnt/β-catenin pathway ↓	[277]	Human	miR-21 ↑ → (RAS/PI3K/AKT pathway ↓ under shear stress; suppression reverses this)	[278]	Human (fetuses via umbilical cord blood)	miR-1 ↓, miR-208 ↓, miR-499 ↓	[279]
Human	miR-21 ↑	[280]	Human	miR-1, miR-133 → KCNJ2, FBN2, SLC38A3, TNNI1	[281]	Mouse and Human (CHD-related RVF tissue)	miR-34a ↑ → HIF-1α, VEGFA, VEGFB, VEGFR2, SIRT1 ↓	[282]
Human (infants with and without TOF)	miR-421 ↑ → SOX4 (Notch signaling) ↓	[283]	Human	22 miRNAs (mostly ↓) → mGLUR, Gq, PLC, PKC, ECM, FAK, PI3K, PDK1 ↑	[284]	Human	miR-8078 ↑ (targets implicated in MAPK cascade, ion transport, atrioventricular valve morphogenesis)	[285]

↑ indicates upregulation/activation, ↓ indicates downregulation/inhibition and → indicates a causal or sequential relationship (“leads to” or “results in”).

## Data Availability

No new data were created or analyzed in this study. Data sharing is not applicable to this article.
